# A practical assembly guideline for genomes with various levels of heterozygosity

**DOI:** 10.1093/bib/bbad337

**Published:** 2023-10-05

**Authors:** Takako Mochizuki, Mika Sakamoto, Yasuhiro Tanizawa, Takuro Nakayama, Goro Tanifuji, Ryoma Kamikawa, Yasukazu Nakamura

**Affiliations:** Genome Informatics Laboratory, National Institute of Genetics; Genome Informatics Laboratory, National Institute of Genetics; Genome Informatics Laboratory, National Institute of Genetics; Division of Life Sciences Center for Computational Sciences, University of Tsukuba, Japan; Department of Zoology, National Museum of Nature and Science; Graduate School of Agriculture, Kyoto University; Genome Informatics Laboratory, National Institute of Genetics

**Keywords:** de novo assembly, genome sequencing, long-read sequencing, heterozygosity, purging allelic sequences, assembly evaluation

## Abstract

Although current long-read sequencing technologies have a long-read length that facilitates assembly for genome reconstruction, they have high sequence errors. While various assemblers with different perspectives have been developed, no systematic evaluation of assemblers with long reads for diploid genomes with varying heterozygosity has been performed. Here, we evaluated a series of processes, including the estimation of genome characteristics such as genome size and heterozygosity, *de novo* assembly, polishing, and removal of allelic contigs, using six genomes with various heterozygosity levels. We evaluated five long-read-only assemblers (Canu, Flye, miniasm, NextDenovo and Redbean) and five hybrid assemblers that combine short and long reads (HASLR, MaSuRCA, Platanus-allee, SPAdes and WENGAN) and proposed a concrete guideline for the construction of haplotype representation according to the degree of heterozygosity, followed by polishing and purging haplotigs, using stable and high-performance assemblers: Redbean, Flye and MaSuRCA.

## INTRODUCTION

The advent of third-generation sequencing technologies, represented by Pacific Biosciences (PacBio) and Oxford Nanopore Technologies (Nanopore), has allowed for very long *de novo* assemblies of complex genomes, including that of eukaryotic diploids [1]. These technologies produce long nucleotide sequence reads by reading long single-molecule nucleic acids [2]. According to the manufacturers’ websites, the average read length and total amount of the read length for PacBio (Sequel, SMRT Cell 1 M) are 30 kb and 20 Gb, respectively, while those for Nanopore (MinION) are up to 4 Mb and 50 Gb, respectively. Therefore, they have been used for *de novo* assembly of challenging genomes with high heterozygosity [3], repetitive regions [4], large size [5] or high ploidy [6]. However, they produce higher sequencing error rates (5–15%) [7] than Illumina short reads (0.3%) [8]. Hence, most recent assemblers have applied the hierarchical approach to correct long-read errors by detecting sequence errors from alignments between long-reads of the same sample before assembly [9]. Even after long-read-based assembly, the assembled sequences are further polished with Illumina short reads [3, 10–13]. Several polishing tools, including Pilon, POLCA and NextPolish, correct sequence errors in the assembled sequences with long reads and/or Illumina short reads [14–16]. Recently, PacBio offered a high-fidelity (HiFi) read technology, which produces an average 13.5 kb [17] of highly accurate (99.9%) reads (https://www.pacb.com/technology/hifi-sequencing/). Consequently, more *de novo* genome assemblers for the HiFi reads were developed [18, 19]. However, since HiFi sequencing is costlier than standard long-read sequencing, genome assembly using conventional long-read technology is preferred [20–22].

*De novo* assemblers for long reads are classified into long-read-only assemblers (e.g. Canu [23], Flye [24], miniasm [25], NextDenovo (https://github.com/Nextomics/NextDenovo) and Redbean [26]) and hybrid assemblers that use short and long reads (e.g. HASLR [27], MaSuRCA [28], Platanus-allee [29], SPAdes [30] and WENGAN [31]). Hybrid assemblers use two methods: (i) First correct long reads with short reads and then assemble with the corrected long reads (e.g. MaSuRCA) and (ii) Assemble short reads into contigs and then construct the scaffolds from the generated contigs with long reads (e.g. HASLR, Platanus-allee, SPAdes and WENGAN). However, obtaining the best-assembled sequences utilizing all the assemblers is challenging owing to the limited computational resources and time spent for analysing, despite the use of cluster servers.

Although *de novo* assemblers have been developed for diploids, including FALCON Unzip [32], Canu, Platanus-allee and Platanus [33], diploid genome assembly remains challenging. The contig set from *de novo* assembly, composed of one sequence pattern between homologous chromosomes and the sequence of the hemizygous region, is the desired haploid representation. However, highly heterozygous regions are not recognized as homologous regions of two chromosomes by any assemblers and are thus assembled separately. Therefore, the assembly size will be larger than the actual genome size [34]. Some tools that distinguish allelic sequences (haplotigs) from homologous regions have been developed to solve this problem, including Purge Haplotig [34], purge_dups [35] and HaploMerger2 [36].

We evaluated assemblers based on computer resource usage (execution time and memory usage), continuity, and completeness using six genomes with various heterozygosity levels and proposed a concrete guideline for the construction of haplotype representation according to the degree of heterozygosity. The optimal genome-assembler combination is influenced by heterozygosity, repeats, genome size, as well as research purposes. Therefore, our guidelines are intended to help users select and further modify the best method to suit their genome characteristics and research purposes.

## MATERIALS AND METHODS

### Collecting the datasets for evaluation

Since we focused on how heterozygosity affects genome assembly, we collected sets of genomes with varying levels of heterozygosity. The six available genomes of *Arabidopsis thaliana* accession C24 [10], *Nitzschia putrida* strain NIES-4239 [37], *Lates calcarifer* [38], *Solanum sitiens* [11], *A. thaliana* F1 cross of Col-0 and Cvi-0 [32] and *Crassostrea gigas* [3] were used. Their PacBio subreads and Illumina paired-end data of whole-genome sequencing were downloaded from the public archive Sequence Read Archive of European Nucleotide Archive (ENA) [39] (Table 1). The sequencing data of the six genomes have PacBio coverage ≥39x and Illumina coverage ≥60x. For the *L. calcarifer* PacBio subreads, 223x out of 247x were retrieved since they are sufficient to gain PacBio coverage >39x.

**Table 1:** Summary of input datasets

Organism	Heterozygosity (%)	Genome size (Mb)	Repeat (%)	BioSample	PacBio coverage (X)	Illumina coverage (X)	Illumina trimmed coverage (X)
*A. thaliana* C24	0.055	125	15.8	SAMEA5751959	49	60	58
*N. putrida* NIES-4239	0.336	32	8.7	SAMD00232021	39	3082	2940
*Lates calcarifer*	0.479	552	2.8	SAMN04026617	223^a^	102	94
*S. sitiens*	0.847	906	43.9	SAMN14932980	56	190	178
*A. thaliana* F1 cross of Col-0 and Cvi-0	1.040	119	13.3	SAMN04539663	332	68 (26)^b^	66
*C. gigas*	3.000	487	28.6	SAMEA6259236	112	82	77

^a^The following no data were used for *de novo* assembly, as the amount of data was sufficient. (SRR3224582, SRR3224583, SRR3224584, SRR3224585, SRR3224586, SRR3224587, SRR3224588, SRR3224589, SRR3224590, SRR3224591, SRR3224593, SRR3224595, SRR3224596, SRR3224597, SRR3224598, SRR3224599, SRR3224602, SRR3224603, SRR3224604, SRR3224605, SRR3224606, SRR3224607, SRR3224608, SRR3224609, SRR3224610, SRR3224612, SRR3224615, SRR3224617, SRR3224618, SRR3224619, SRR3224620, SRR3224621, SRR3224622, SRR3224623, SRR3224624, SRR3224625, SRR3224626, SRR3224627, SRR3224628, SRR3224629, SRR3224630, SRR3224631, SRR3224632, SRR3224633, SRR3224634, SRR3224635, SRR3224636, SRR3224637, SRR3224638, SRR3224639, SRR3224640, SRR3224641, SRR3224642, SRR3224643, SRR3224644, SRR3224645, SRR3224646, SRR3224647, SRR3224648, SRR3224649, SRR3224650, SRR3224651, SRR3224652, SRR3224653, SRR3224654, SRR3224655, SRR3224656, SRR3224657, SRR3224658, SRR3224659, SRR3224660, SRR3224661, SRR3224662, SRR3224663, SRR3224664, SRR3224665, SRR3224666, SRR3224667, SRR3224668, SRR3224669, SRR3224670, SRR3224671, SRR3224672, SRR3224673, SRR3224674, SRR3224675, SRR3224676, SRR3224677, SRR3224678, SRR3224679, SRR3224680, SRR3224681, SRR3224682, SRR3224683, SRR3224684, SRR3224685, SRR3224686)

^b^Illumina data in SRA of *A. thaliana* F1 include reads with a variety of lengths. Therefore, only reads that were 250 bp length were extracted and used for the MaSuRCA assembler.

### Analytical processes

**Figure 1:**
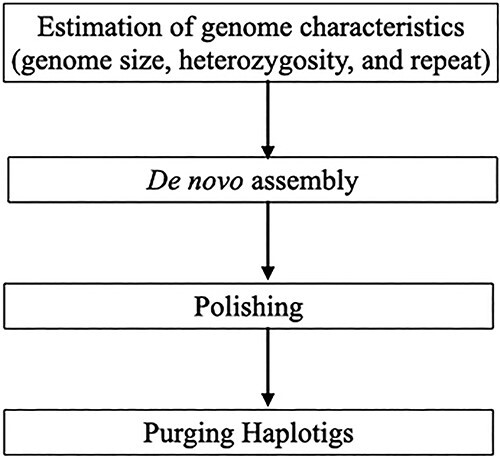
Evaluated analytical processes.

The stepwise analytical processes for the construction of haploid representation comprised four processes: estimation of genome characteristics (such as genome size, heterozygosity, and repeat rate), *de novo* assembly, polishing, and purging of haplotigs (Figure 1). First, the genome characteristics were estimated by obtaining the k-mer counts through Jellyfish v2.2.10 [40] (-C -m 21 -s 1000000000) and using them to estimate the genome characteristics through GenomeScope [41] (k-mer_length = 21 and kmermax = 1000). The estimated genome characteristics were assessed by comparing the genome size with that in the original research.

*De novo* assembly was then conducted. Illumina paired-end reads used for the hybrid assembler were trimmed using fastp v0.20.0 [42] (—cut_front —cut_tail option). Thereafter, the assembled sequences that were > 500 b in length were extracted using seqkit v0.15.0 [43]. To polish the extracted assembled sequences, Illumina paired-end reads trimmed in the previous step were mapped to the assembled sequences using bwa v2.2.1 [44], and the result was determined using Pilon v1.24 [14]. The manipulation of SAM/BAM files was performed using SAMtools v1.11 [45]. Then, the haplotigs were removed from the polished sequences through Purge Haplotigs v1.1.1 [34]. The read-depth cut-off parameters for the ‘purge’ command of Purge Haplotigs comprised three types, i.e. ‘low cut-off’ for removing assembly artefacts, ‘midpoint’ for specifying between the haploid and diploid peaks, and ‘high cut-off’ for removing repeats and organelles. These were set (Supplementary Table S1) by referring to the histograms of read-depth to contigs (Supplementary Figure S1) generated using the ‘hist’ command of Purge Haplotigs. Purge Haplotigs outputs three types of FASTA format files: haploid representation (primary sequences), haplotigs and artefacts (comprising assembly artefacts, organelle genome and repeat sequences). The details of the execution commands and configuration options of each tool during the *de novo* assembly and thereafter are described in the Supplementary Methods. The graph of cumulative length and Nx statistics for contigs were generated using R ver. 4.0.5. Assembly ploidy, a metric that estimates the proportion of allelic sequences present in the assembled genome sequences, was calculated by dividing the total length by the estimated genome size [8].

### *De novo* assemblers

Long-read-only assemblers were Canu (v2.1.1) [23], Flye (v2.8.3) [24], miniasm (v0.3-r179) [25], NextDenovo (v2.4.0) (https://github.com/Nextomics/NextDenovo), and Redbean (v2.5) [26] with PacBio long reads. Hybrid assemblers were HASLR (v0.8a1) [27], MaSuRCA (v4.0.1) [28], Platanus-allee (v2.2.2) [29], SPAdes (v3.15) [30] and WENGAN (v0.2) [31] with both PacBio long reads and Illumina paired-end reads. MaSuRCA and WENGAN assemble using external *de novo* assemblers. We benchmarked using MaSuRCA_C (CABOG [46]), MaSuRCA_F (Flye) and WENGAN-M (MINIA3 [47]). These long-read-only and hybrid assemblers are summarized in the Supplementary Notes. The assemblers that finished successfully among all six genomes were selected for evaluation. If the execution time exceeded 500 h, it was evaluated as ‘time out’ and not subjected to subsequent analysis or comparison, regardless of the final result.

Platanus-allee generates phased sequences and consensus sequences that are not phased. The other assemblers generate consensus sequences. Herein, the consensus sequences were consistently used for comparison among assemblers. Additionally, Flye, MaSuRCA_F and Platanus-allee output scaffolds, whereas SPAdes and MaSuRCA_C output scaffolds and/or contigs. The other assemblers output contigs but not scaffolds. Therefore, for comparison, we utilized scaffolds from Flye, MaSuRCA_F, Platanus-allee, SPAdes and MaSuRCA_C, as well as contigs from the other tools. Hereafter, both scaffolds and contigs are referred to as ‘contigs’ without distinction.

### Evaluation of *de novo* assemblers

The resultant contigs were evaluated based on continuity and completeness. Contig continuity was evaluated by N50, which is the contig length when 50% of the total contig size is reached, while the assembled contig lengths were added in the longest order. The statistics of assembled contigs were calculated using assembly-stats v1.0.1 (https://github.com/rjchallis/assembly-stats) and Merqury v1.3 [48]. The completeness was measured using Benchmarking Universal Single-Copy Orthologs (BUSCO) analysis ver. 5.0.0 [49]. BUSCO databases used ‘embryophyta_odb10’ for *A. thaliana* C24 and F1, ‘eukaryota_odb10’ for *N. putrida* NIES-4239, ‘actinopterygii_odb10’ for *L. calcarifer*, ‘solanales_odb10’ for *S. sitiens*, and ‘metazoa_odb10’ for *C. gigas*. The N50 and BUSCO completeness of polished contigs were ranked for each genome as the best to third best and the worst to third worst. The worst ranking for BUSCO completeness was only used for the percentage of BUSCO completeness, including ‘single-copy’ and ‘duplicated’ if there was a difference of 15 or more from the top value in each genome. The assembler that reached the timeout was ranked as the worst for both N50 and BUSCO completeness. The continuity and completeness scores for each tool were calculated separately for heterozygosity <1 and heterozygosity ≥1 by adding 3 to the best, 2 to second best, 1 to third best, −3 to worst, −2 to second worst, and − 1 to third worst. Based on these scores, the performance of BUSCO completeness and N50 for each assembler was classified as ‘high’, ‘medium’ or ‘low’. The thresholds for each classification were as follows. For heterozygosity <1: high ≥5, 4 ≥ medium ≥ −4 and low ≤ −5 for N50; high ≥5, 4 ≥ medium ≥1 and low ≤0 for BUSCO. For heterozygosity ≥1: high ≥3, 2 ≥ medium ≥ −2, and low ≤ −3 for N50; high ≥4, 3 ≥ medium ≥0 and low ≤ −1 for BUSCO. These classifications of the assemblers by computational resource usage, N50 and BUSCO completeness were used to select the assemblers in the guideline adapted to the degree of heterozygosity. Among the assemblers with similar evaluation, the assembler with the most stable performance for N50 and BUSCO completeness was adopted.

To measure computational usage under the same conditions, all *de novo* assemblers were utilized on the National Institute of Genetics supercomputer system medium nodes with 10 CPU cores (CPU: Intel Xeon Gold 6148 × 4, 80 core/node). To measure wall-clock time and memory usage, ‘ru_wallclock’ and ‘maxvmem’ reported by the qacct command of the Univa Grid Engine were used. The assemblers were classified as ‘Light’, ‘Medium’ or ‘Heavy’ according to their maximum values of the wall-clock time and memory usage. The classification thresholds for the maximum value of wall-clock time for each tool were Light <50 h, 50 h ≤ Medium <250 h and Heavy ≥250 h. The classification thresholds for the maximum value of memory usage for each tool were Light <50 GB, 50 GB ≤ Medium <400 GB and Heavy ≥400 GB. The assemblers were classified by computational resource usage, including the wall-clock time and memory usage, as a comprehensive evaluation. The thresholds for ‘Light’ and ‘Heavy’ were the maximum value of wall-clock time < 50 h with the maximum value of memory usage <300 GB, and the maximum value of wall-clock time ≥ 250 h or the maximum value of memory usage ≥400 GB, respectively. ‘Medium’ was defined as other than Light or Heavy in this case.

Furthermore, the contigs of *Arabidopsis* F1 hybrids were evaluated by comparing with the parental haploid sequences, Col-0 (TAIR10) and Cvi-0 [10], using QUAST v5.0.2 [50]. Genome fraction is the percentage of the total number of bases aligned with contigs divided by the reference genome size. NGA50 is similar to N50 but uses the alignment block length and reference genome length instead of the contig length and total contig size for N50. The alignment block lengths are calculated by splitting contigs at misassembly breakpoints. Thus, NGA50 is the alignment block length at 50% of the total reference genome size.

## RESULTS

### Estimating genome characteristics

GenomeScope was used for all six genomes, and the estimated genome sizes were compared to the original research (Supplementary Table S2). The differences of estimated genome sizes from those of original research ranged from 6 to 27% in GenomeScope. The genome statistics estimated by GenomeScope were 0.055–3.00% heterozygosity, 32–906 Mb genome size and 2.8–43.9% repeats (Table 1).

### Assembly and polishing evaluation

#### Effect of polishing on assembly continuity

Contig statistics of assembly contigs and polished contigs are described in Supplementary Table S3 and Supplementary Table S4, respectively. Even after polishing, the total lengths obtained by almost all the assemblers did not change much from those before polishing (Supplementary Table S5). However, the largest contig length of miniasm for *L. calcarifer* decreased by 1.84% after polishing. Similarly, after polishing, the total contig length of miniasm for *N. putrida* NIES-4239 decreased by 1.59% after polishing and that of N50 of miniasm for *L. calcarifer* decreased by 2.58%. Therefore, polishing has a certain impact on contig length assembly, and we compared the assembly ploidy as well as continuity among the assemblers with polished contigs.

#### Assembly ploidy and continuity after polishing

**Figure 2:**
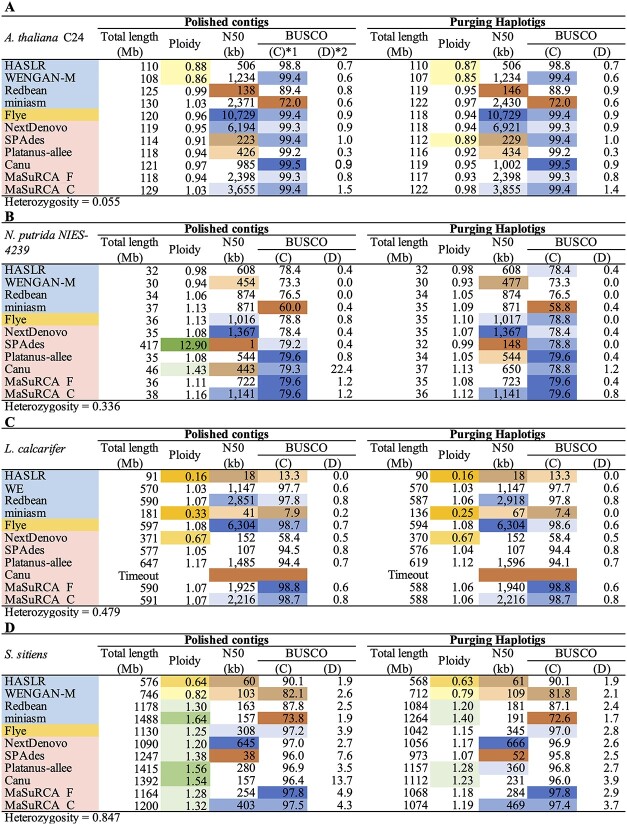

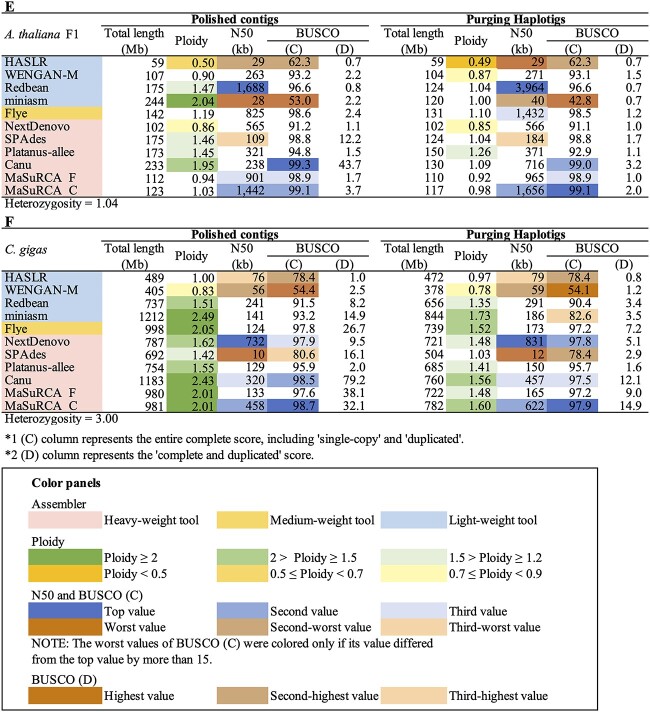
Statistics and BUSCO completeness of polishing and purging haplotigs. (A) *A. thaliana* C24; (B) *N. putrida* NIES-4239; (C) *Lates calcarifer*; (D) *S. sitiens*; (E) *A. thaliana* F1; (F) *C. gigas*. Ploidy column represents ‘assembly ploidy’. BUSCO (C) column represents the entire complete score, including ‘single-copy’ and ‘duplicated’. BUSCO (D) column represents the ‘complete and duplicated’ score.

As heterozygosity increased, the assembly ploidy of each assembler also increased (Figure 2). The graphs of the cumulative length for contigs are shown in Supplementary Figure S2 and the concrete values of assembly size in Supplementary Table S4. As for the genomes with heterozygosity <0.5, including those of *A. thaliana* C24, *N. putrida* NIES-4239, and *L. calcarifer*, most assemblers show that the assembly ploidies are approximately 1, implying that the total contig sizes almost equal the estimated genome sizes. However, in the *S. sitiens* genome (heterozygosity, 0.847), the assembly ploidy in miniasm, Platanus-allee and Canu exceeded 1.5. In the *A. thaliana* F1 genome (heterozygosity, 1.04), miniasm and Canu showed an assembly ploidy of approximately 2. Furthermore, in the *C. gigas* genome (heterozygosity, 3.00), the assembly ploidy in miniasm, Flye, Canu, MaSuRCA_F and MaSuRCA_C exceeded 2, whereas that in Redbean, NextDenovo, and Platanus-allee exceeded 1.5. In the *N. putrida* NIES-4239 genome (heterozygosity, <0.5), SPAdes showed a much larger assembly ploidy of 12.90, whereas that of HASLR and WENGAN-M tended to be smaller overall.

The values of N50 are indicated in Figure 2; the graphs of the Nx statistics for contigs, in Supplementary Figure S3; and the concrete statistical values, including number of contigs, largest contig length, total length, N50, number of Ns and number of gaps, are listed in Supplementary Table S4. The continuity score, which is obtained by ranking N50 of each genome and summing the values of each rank, calculated for genomes with a heterozygosity <1 and ≥ 1, are indicated in Tables 2A and B, respectively. Consequently, for genomes with a heterozygosity <1, Flye, NextDenovo and MaSuRCA_C were classified as ‘high’; MaSuRCA_F, Platanus-allee, Redbean, miniasm, WENGAN_M and HASLR as ‘medium’; and Canu and SPAdes as ‘low’. For genomes with a heterozygosity ≥1, MaSuRCA_C, NextDenovo and Redbean were classified as ‘high’; Canu, MaSuRCA_F, Flye, Platanus-allee and WENGAN_M as ‘medium’; and HASLR, miniasm and SPAdes as ‘low’. In general, MaSuRCA_C and NextDenovo provided better continuity regardless of heterozygosity in the given genomes, while Flye provided better continuity for genomes with a heterozygosity <1.

**Table 2:** Comprehensive evaluation of *de novo* assemblers A. Continuity and BUSCO completeness (Heterozygosity <1) B. Continuity and BUSCO completeness (Heterozygosity ≥1) C. Computational resource usage

A			
Tool	Computer usage	Continuity	BUSCO completeness
HASLR	Light-weight tool	Medium (−4)	Low (−1)
WENGAN-M	Light-weight tool	Medium (−2)	Low (0)
Redbean	Light-weight tool	Medium (−1)	Medium (1)
miniasm	Light-weight tool	Medium (−1)	Low (−11)
Flye	Medium-weight tool	High (8)	High (5)
NextDenovo	Heavy-weight tool	High (8)	Medium (1)
SPAdes	Heavy-weight tool	Low (−8)	Medium (3)
Platanus-allee	Heavy-weight tool	Medium (−1)	Medium (3)
Canu	Heavy-weight tool	Low (−5)	Medium (2)
MaSuRCA_F	Heavy-weight tool	Medium (0)	High (10)
MaSuRCA_C	Heavy-weight tool	High (6)	High (9)
**B**			
**Tool**	**Computer usage**	**Continuity**	**BUSCO completeness**
HASLR	Light-weight tool	Low (−3)	Low (−4)
WENGAN-M	Light-weight tool	Medium (−2)	Low (−3)
Redbean	Light-weight tool	High (3)	Medium (0)
miniasm	Light-weight tool	Low (−3)	Low (−3)
Flye	Medium-weight tool	Medium (0)	Medium (0)
NextDenovo	Heavy-weight tool	High (3)	Medium (1)
SPAdes	Heavy-weight tool	Low (−4)	Low (−1)
Platanus-allee	Heavy-weight tool	Medium (0)	Medium (0)
Canu	Heavy-weight tool	Medium (1)	High (5)
MaSuRCA_F	Heavy-weight tool	Medium (1)	Medium (1)
MaSuRCA_C	Heavy-weight tool	High (4)	High (5)
**C**			
**Tool**	**Computer usage**	**Wall-clock time**	**Memory intensity**
HASLR	Light-weight tool	Light	Light
WENGAN-M	Light-weight tool	Light	Light
Redbean	Light-weight tool	Light	Medium
miniasm	Light-weight tool	Light	Medium
Flye	Medium-weight tool	Medium	Medium
NextDenovo	Heavy-weight tool	Heavy	Medium
SPAdes	Heavy-weight tool	Heavy	Heavy
Platanus-allee	Heavy-weight tool	Heavy	Heavy
Canu	Heavy-weight tool	Heavy	Heavy
MaSuRCA_F	Heavy-weight tool	Heavy	Heavy
MaSuRCA_C	Heavy-weight tool	Heavy	Heavy

### Effect of polishing on annotation completeness (BUSCO)

**Figure 3:**
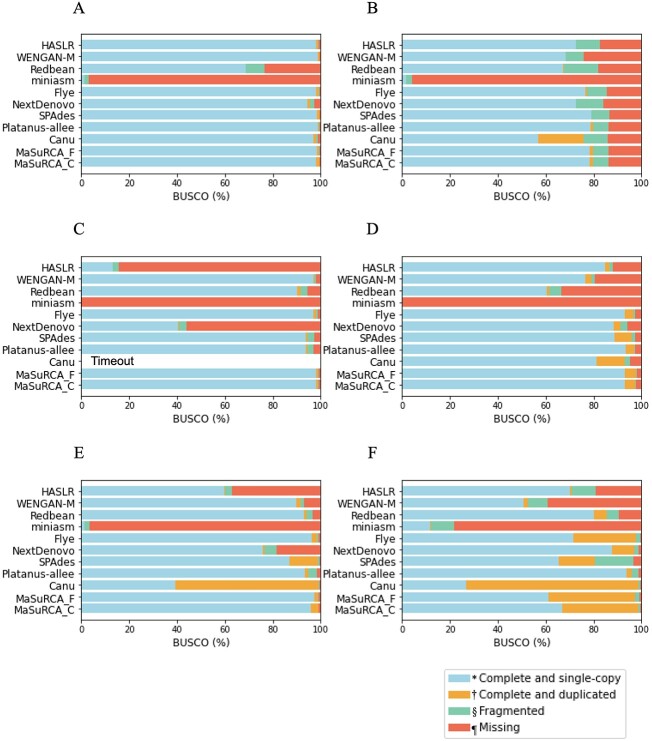
BUSCO completeness for assembly contigs (A) *A. thaliana* C24; (B) *N. putrida* NIES-4239; (C) *Lates calcarifer*; (D) *S. sitiens*; (E) *A. thaliana* F1; (F) *C. gigas.* Each breakdown and colour bar represent the various BUSCO categories: the entire complete score, including ‘single-copy’ and ‘duplicated’, is represented by ‘C’. ‘*’ shows ‘single-completeness’, represented by ‘S’. ‘†’ shows ‘duplicated-completeness’, represented by ‘D’. ‘§’ shows ‘Fragmented’, represented by ‘F’. ‘¶’ shows ‘Missing’, represented by ‘M’. Canu was timeout in (C).

**Figure 4:**
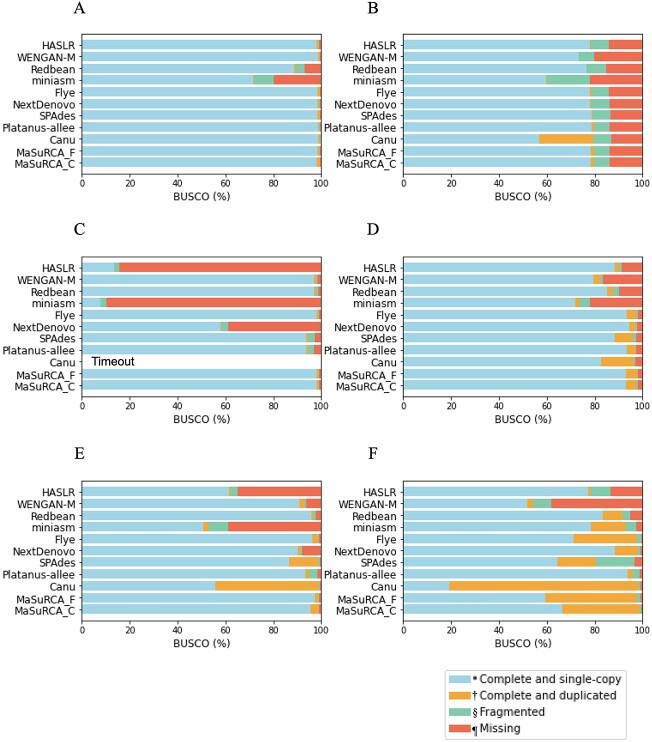
BUSCO completeness for the polished contigs (A) *A. thaliana* C24; (B) *N. putrida* NIES-4239; (C) *Lates calcarifer*; (D) *S. sitiens*; (E) *A. thaliana* F1; (F) *C. gigas.* Each breakdown and colour bar represent the categories of BUSCO: the entire complete score, including ‘single-copy’ and ‘duplicated’, is represented by ‘C’. ‘*’ shows ‘single-completeness’, represented by ‘S’. ‘†’ shows ‘duplicated-completeness’, represented by ‘D’. ‘§’ shows ‘Fragmented’, represented by ‘F’. ‘¶’ shows ‘Missing’, represented by ‘M’. Canu was timeout in (C).

BUSCO completeness of assembly contigs and polished contigs are shown in Figures 3 and 4, respectively. The concrete BUSCO scores are indicated in Supplementary Table S6 and Supplementary Table S7. In particular for miniasm and Redbean, BUSCO completeness tended to improve significantly after polishing (Supplementary Table S8). For SPAdes, MaSuRCA_F and MaSuRCA_C, there was little change. Platanus-allee remained unchanged in all genomes. For HASLR, WENGAN-M, Flye, NextDenovo and Canu, the BUSCO completeness moderately improved after polishing. Since these results indicated that BUSCO completeness improved significantly before and after polishing the assembled contigs, especially with miniasm and Redbean, BUSCO completeness among the assemblers was compared based on the results after polishing in the following section.

### Effect of heterozygosity on annotation completeness (BUSCO)

The ‘complete score’ (C) column and ‘complete and duplicate score’ (D) column of BUSCO for after polishing are indicated in Figure 2. BUSCO completeness after polishing is shown in Figure 4 and Supplementary Table S7. The genomes of *A. thaliana* C24 (heterozygosity, 0.055) and *N. putrida* NIES-4239 (heterozygosity, 0.336), which have lower heterozygosity, showed that their BUSCO completeness was not noticeably different among the assemblers, except for miniasm. For the other genomes, such as *L. calcarifer* (heterozygosity, 0.479), the BUSCO completeness was explicitly different among the assemblers, from 7.9 in miniasm to 98.8 in MaSuRCA_F. The completeness score, which is obtained by ranking the scores for complete BUSCO genes of each genome and summing the values of each rank, for each tool was separately calculated for genomes with heterozygosity <1 and ≥ 1 (Tables 2A and B). Consequently, for genomes with a heterozygosity <1, Flye, MaSuRCA_F and MaSuRCA_C were classified as ‘high’; SPAdes, Platanus-allee, Canu, Redbean and NextDenovo as ‘medium’; and WENGAN-M, HASLR and miniasm as ‘low’. For genomes with a heterozygosity ≥1, Canu and MaSuRCA_C were classified as ‘high’; NextDenovo, MaSuRCA_F, Redbean, Flye and Platanus-allee as ‘medium’; and SPAdes, WENGAN-M, miniasm and HASLR as ‘low’. Across the various levels of heterozygosity, MaSuRCA_F, MaSuRCA_C, Flye and Canu provided stable and high BUSCO completeness. The complete and duplicated BUSCO scores were higher in most assemblers for genomes with a heterozygosity >0.5 (e.g. *S. sitiens*, *A. thaliana* F1, and *C. gigas*) than for the other genomes; that of Canu was particularly large.

#### Computational resource usages

**Figure 5:**
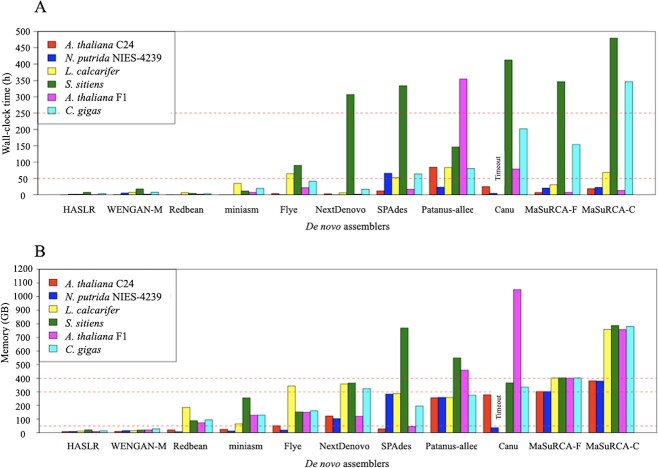
Computational resource usage of *de novo* assemblers. Canu for *Lates calcarifer* timed-out. See Supplementary Table S9 for actual wall-clock time and memory. (A) Wall-clock time in hours. Two horizontal dashed lines indicate 50 and 250 h. (B) Memory usage. Three horizontal dashed lines indicate 50, 300 and 400 GB.

Computational resource usages in the *de novo* assembly process are represented in Figure 5 and the concrete numeric values, in Supplementary Table S9. The maximum wall-clock time values exceeded 250 h for NextDenovo, SPAdes, Platanus-allee, Canu, MaSuRCA_F and MaSuRCA_C, whereas that of HASLR, WENGAN-M, Redbean and miniasm did not exceed 50 h. Flye had a maximum wall-clock time of 90 h. The wall-clock time of Canu of *L. calcarifer* exceeded 500 h and became a ‘timeout’. Subsequently, to evaluate the wall-clock times, HASLR, WENGAN-M, Redbean and miniasm were classified as ‘Light’; Flye, as ‘Medium’; and NextDenovo, SPAdes, Platanus-allee, Canu, MaSuRCA_F and MaSuRCA_C, as ‘Heavy’ (Table 2C).

The maximum value of memory usage exceeded 400 GB for SPAdes, Platanus-allee, Canu, MaSuRCA_F and MaSuRCA_C. Neither HASLR nor WENGAN-M used >50 GB of memory in any condition. The maximum memory usages of Redbean and miniasm were both <300 GB but >50 GB. The maximum memory usages for Flye and NextDenovo were 343 and 364 GB, respectively. Consequently, to evaluate memory usage, HASLR and WENGAN-M were classified as ‘Light’; Redbean, miniasm, Flye and NextDenovo, as ‘Medium’; and SPAdes, Platanus-allee, Canu, MaSuRCA_F and MaSuRCA_C, as ‘Heavy’ (Table 2C).

We then classified the *de novo* assemblers based on the computational resource usage comprising the wall-clock time and the maximum memory usage. HASLR, WENGAN-M, Redbean and miniasm were classified as ‘Light’; Flye, as ‘Medium’; and NextDenovo, SPAdes, Platanus-allee, Canu, MaSuRCA_F and MaSuRCA_C, as ‘Heavy’. Hereinafter, assemblers are referred to as ‘light-weight tool’, ‘medium-weight tool’ or ‘heavy-weight tool’ according to the categories above based on their computational resource usage.

### Evaluation of haplotig removal

#### Assembly ploidy

After the execution of Purge Haplotigs, assembly ploidies in most of the assemblers were closer to 1 across the genomes (Figure 2 and Supplementary Figure S2). Although there was no assembly with ploidy over 2, only miniasm, Flye, Canu and MaSuRCA_C provided an assembly ploidy >1.5 for genomes with higher heterozygosities, such as for *C. gigas* (heterozygosity, 3.00), suggesting difficulty in haplotype removal for these genomes. The concrete statistical values for primary contigs, haplotigs, and artefacts are indicated in Supplementary Table S10.

#### Annotation completeness (BUSCO)

The output files from Purge Haplotigs, primary contigs, haplotigs, and artefacts were examined for BUSCO completeness (Supplementary Figures S4–S6) to evaluate the validity of the purging process. The contig sets with higher complete and duplicated BUSCO scores were purged well (Figure 2). The highest BUSCO duplicated score before purging was 79.2% in Canu of *C. gigas*; after purging, this score drastically decreased to 12.1%. The BUSCO duplicated scores of *C. gigas*, which were higher in any of the assemblers than the other genomes, decreased to 14.9% after purging for MaSuRCA_C at the highest. In contrast, the BUSCO completeness of primary contigs was similar to that before the removal of haplotigs from across the genomes (Supplementary Table S11). The completeness scores that decreased by >1% were those of miniasm for *N. putrida* NIES-4239 (heterozygosity, 0.336); miniasm for *S. sitiens* (heterozygosity, 0.847), miniasm and Platanus-allee for *A. thaliana* F1 (heterozygosity, 1.04); and Redbean, miniasm, SPAdes and Canu for *C. gigas* (heterozygosity, 3.00). The maximum decreased score was 10.6% of miniasm for *C. gigas*. That is, decrease in BUSCO completeness >1% was not observed even after Purge Haplotigs for any genomes in HASLR, WENGEN-M, Flye, NextDenovo, MaSuRCA_F or MaSuRCA_C.

These results suggest that some might have been over-purged. To survey the over-purge, we examined (i) the number of BUSCO genes that were detected on haplotigs but not on primary contigs and (ii) the number of BUSCO genes that were detected on artefacts but not on primary contigs (Supplementary Table S12). Consequently, an over-purge >1% of the total BUSCO genes was not observed in any of the assembled genomes with HASLR, WENGEN-M, Flye, NextDenovo, MaSuRCA_F or MaSuRCA_C. Conversely, for genomes with a heterozygosity >0.847 (such as that for *S. sitiens*), an over-purge was observed in some assemblers. The maximum number of (A) was 160 in miniasm for *A. thaliana* F1 (9.9% of the overall BUSCO genes), and that of (B) was 10 in Platanus-allee for *A. thaliana* F1 (0.6% of the overall BUSCO genes).

#### Comparison with Arabidopsis reference genome

To evaluate how effectively the above processes (assembly, polishing and purging) reconstructed the haploid sequences, the assembled *A. thaliana* F1 primary contigs were compared with the haploid sequences of Col-0 and Cvi-0, which are parent accessions (Supplementary Table S13). The genome fractions, NGA50 values, or the number of misassemblies between F1 and Col-0 and between F1 and Cvi-0 were comparable. For Redbean, Flye, SPAdes, Platanus-allee, Canu and MaSuRCA_C, the genome fractions were > 90%, while they were 88% for MaSuRCA_F and approximately 80% for NextDenovo and WENGAN-M. However, in miniasm and HASLR, the genome fractions were 52% and < 50%, respectively. The NGA50 values in Redbean, Flye, Canu, MaSuRCA_F and MaSuRCA_C were > 130 Kb; miniasm had the lowest NGA50 value (approximately 3000 b). The NGA50 values of the remaining assemblers were almost 8000–12 000 b. HASLR has no NGA50 value because the total aligned length is <50% of the length of the parent reference genome. While the number of misassemblies was the smallest in HASLR (approximately 450), that in the others was 1750–4722, with that in Platanus-allee being the highest.

#### Analytical guideline

**Figure 6:**
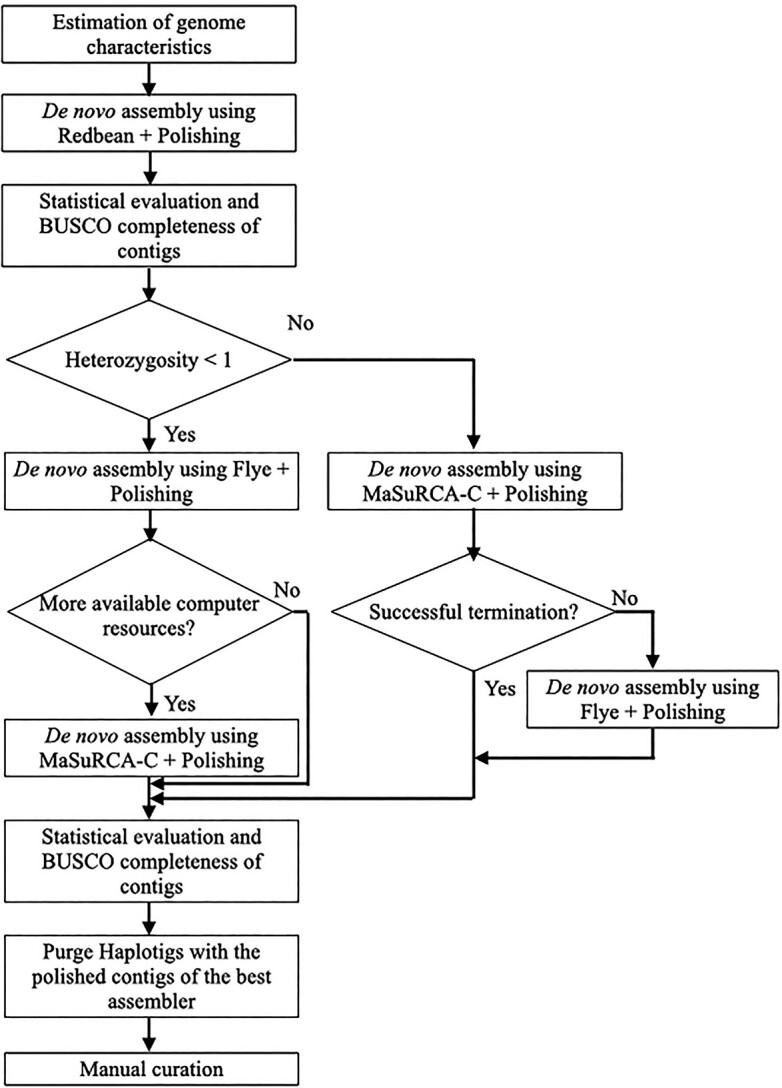
Recommended analytical guideline. This guideline consists of four processes: sample properties estimation, *de novo* assembly, polishing and purging haplotigs. *De novo* assemblers are recommended separately for heterozygosities <1 and ≥ 1.

We devised a guideline to construct a haploid representation with PacBio long reads and Illumina short reads for diploid genomes with various levels of heterozygosity (Figure 6). This was based on the evaluation of the continuities and BUSCO scores for each process of assembling, polishing and purging haplotigs using various assemblers. First, to understand the sample properties such as genome size, the heterozygosity and repeat rate are estimated using tools such as GenomeScope. For evaluating *de novo* assemblers, it is recommended to use only polished contigs after assembly. For genomes with any heterozygosities, the first recommended assembler is Redbean, a light-weight tool (Table 2C) with a stable performance regarding continuity and BUSCO completeness, regardless of heterozygosity (Table 2A and B). Redbean can provide a rough indication of computational resource usage, continuity and BUSCO completeness when using other additional assemblers. For genomes with a heterozygosity <1, Flye can be used as the second trial assembler because it is a medium-weight tool (Table 2C) classified as ‘High’ for both continuity and BUSCO completeness when heterozygosity is <1 (Table 2A). If memory and execution time are more available than usage for Flye, MaSuRCA_C should be used because it is a heavy-weight tool (Table 2C) classified as ‘High’ for both continuity and BUSCO completeness when heterozygosity is <1 (Table 2A). For genomes with a heterozygosity ≥1, MaSuRCA_C should be used as the alternative second trial assembler because it is a heavy-weight tool (Table 2C) classified as ‘High’ both for continuity and BUSCO completeness and has a stable performance across the genomes with any heterozygosity (Table 2A and B). If MaSuRCA_C does not terminate successfully or the execution time is too long, it is better to use Flye as a medium-weight tool even for genomes with a heterozygosity ≥1. Flye is inferior to MaSuRCA_C in both continuity and BUSCO completeness (Table 2B), but it provides stable results with lower computational resources than MaSuRCA_C does (Table 2C). If two or more assemblers are used, their continuity and BUSCO completeness must be compared. Finally, removal of haploid duplication is performed using tools such as Purge Haplotigs. This process is more important for genomes with a higher heterozygosity. After purging, the results need to be verified, and manual curation is required.

## DISCUSSION

Herein, we evaluated the procedures for constructing a haploid representation from PacBio long reads and Illumina short reads by focusing on heterozygosity and suggested an analytical guideline adapted to the degree of heterozygosity. The guideline includes: (i) estimation of genome information, including genome size and heterozygosity, (ii) *de novo* assembly, (iii) polishing and (iv) removal of duplicated haploid sequences. The five long-read-only and hybrid assemblers, respectively, were assessed for computer resource usage, contig continuity and BUSCO completeness (Table 2). Contig continuity and BUSCO completeness were separately evaluated for genomes with heterozygosities <1 and for those with heterozygosities ≥1. Subsequently, we selected three high-performance assemblers: Redbean from light-weight tools, Flye from medium-weight tools and MaSuRCA_C from heavy-weight tools. These assemblers were incorporated into the analytical guideline (Figure 6).

We focused on heterozygosity and proposed recommended assemblers. Nevertheless, the best genome–assembler combination would be affected by heterozygosity, repeats, genome size and other factors that cannot be determined without practically testing them. Thus, it is essential to understand the features of assemblers and perform a selective trial. We recommend checking the following items to choose the best assembly contigs: (i) BUSCO completeness, (ii) continuity and (iii) comparison between contig size after purging haplotigs and estimated genome size. For (i), this study conducted polishing once. However, polishing iteration may be effective if time permits, particularly for Redbean and miniasm, which showed significant polishing effects. A comparison of assemblers sometimes shows a similar BUSCO completeness value, indicating the limitation of the evaluations using BUSCO completeness. Thus, not only BUSCO but also the mapping rate of RNA-seq and/or Iso-seq might help select the best assembler. For (ii), we evaluated the continuity with N50 metrics. Depending on the purpose of the individual analysis, other metrics such as the number of contigs and largest contig length would be useful. For (iii), if BUSCO completeness, continuity and genome size are less than the expected values even after using multiple tools, a review of the methods, such as DNA quality, may be required prior to assembly.

While purging haplotigs was assessed by focusing on over-purging with the N50 metrics, BUSCO completeness, and assembly polyploidy, under-purging is still a possibility. Therefore, another study [51] evaluated haplotig purging with other metrics along with N50 and BUSCO completeness and a combination of some purging tools. However, evaluation using metrics has limitations. Therefore, to construct an ideal haploid representation, manual curation is needed. Manual curation would be better for genes predicted on the primary contigs, haplotigs and artefacts and to examine whether the predicted genes on the primary contigs cover the predicted genes on the haplotigs or artefacts. Moreover, the removal of organelle genome sequences from the primary and haplotig contigs is required. It is also necessary to remove organelle genomic sequences from the primary and haplotig contigs by homology searches against organelle sequences available at NCBI RefSeq [52].

In summary, this strategy provides a more efficient and improved quality analysis of the diploid genome. Furthermore, this guideline will be useful for beginners in bioinformatics and bioinformaticians involved with challenging genomes.

**Key Points**

- Prior to *de novo* assembly, genomic characteristics (including genome size, heterozygosity, and repeat rate) should be estimated using tools such as GenomeScope to obtain better contigs owing to a better understanding of the genomic characteristics.
- *De novo* assemblers were classified into three groups based on computational resources: Light-weight (HASLR, WENGAN-M, Redbean and miniasm), medium-weight (Flye), and heavy-weight tools (NextDenovo, SPAdes, Platanus-allee, Canu and MaSuRCA).
- *De novo* assemblers were evaluated using N50 and BUSCO completeness for heterozygosity of <1 and ≥ 1. Redbean, Flye and MaSuRCA_C provided high-performance results and were incorporated into the guideline.
- Assembler evaluation using BUSCO completeness requires the use of contigs after polishing because the degree of improvement of BUSCO completeness after polishing varies among assemblers.
- The removal of allelic contigs, which are assembled separately, is necessary to improve the quality of subsequent analyses, such as gene prediction and expression analysis.

## Supplementary Material

Supplementary_Figuresv2_bbad337

Supplementary_Tablesv2_bbad337

Supplementary_Methods_bbad337

Supplementary_Notes_bbad337

## Author Biographies

**Takako Mochizuki** is a research scientist at the Genome Informatics Laboratory, National Institute of Genetics, Japan. Her research interest in bioinformatics includes genome assembly, gene annotation, and structural variation.

**Mika Sakamoto** is a research scientist at the Genome Informatics Laboratory, National Institute of Genetics, Japan. Her research interests in bioinformatics include gene annotation, genetic variation, and protein folding prediction.

**Yasuhiro Tanizawa** is an assistant professor at the Genome Informatics Laboratory, National Institute of Genetics, Japan. His research interests include genome bioinformatics and web services and database development.

**Takuro Nakayama** is an assistant professor at the Division of Life Sciences, Center for Computational Sciences, University of Tsukuba, Japan. His research interests include genome evolutions of single-celled organisms.

**Goro Tanifuji** is a senior curator at the Department of Zoology, National Museum of Nature and Science, Japan. His research interests include genome evolution related to symbiogenesis.

**Ryoma Kamikawa** is an associate professor at Graduate School of Agriculture, Kyoto University, Japan. His research interests include the diversity of microbial eukaryotes.

**Yasukazu Nakamura** is a professor at the Genome Informatics laboratory, National Institute of Genetics, Japan. His research interests include genome informatics.
