# Supplementary material for: A practical assembly guideline for genomes with various levels of heterozygosity
Source: Brief Bioinform. 2023 Oct 5;24(6):bbad337. doi: 10.1093/bib/bbad337 (PMC10555665; doi:10.1093/bib/bbad337)
Supplement: Supplementary_Figuresv2_bbad337 [file supplementary_figuresv2_bbad337.docx]

1. *A. thaliana* C24

HASLR


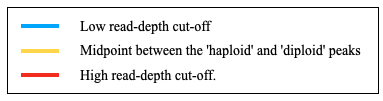


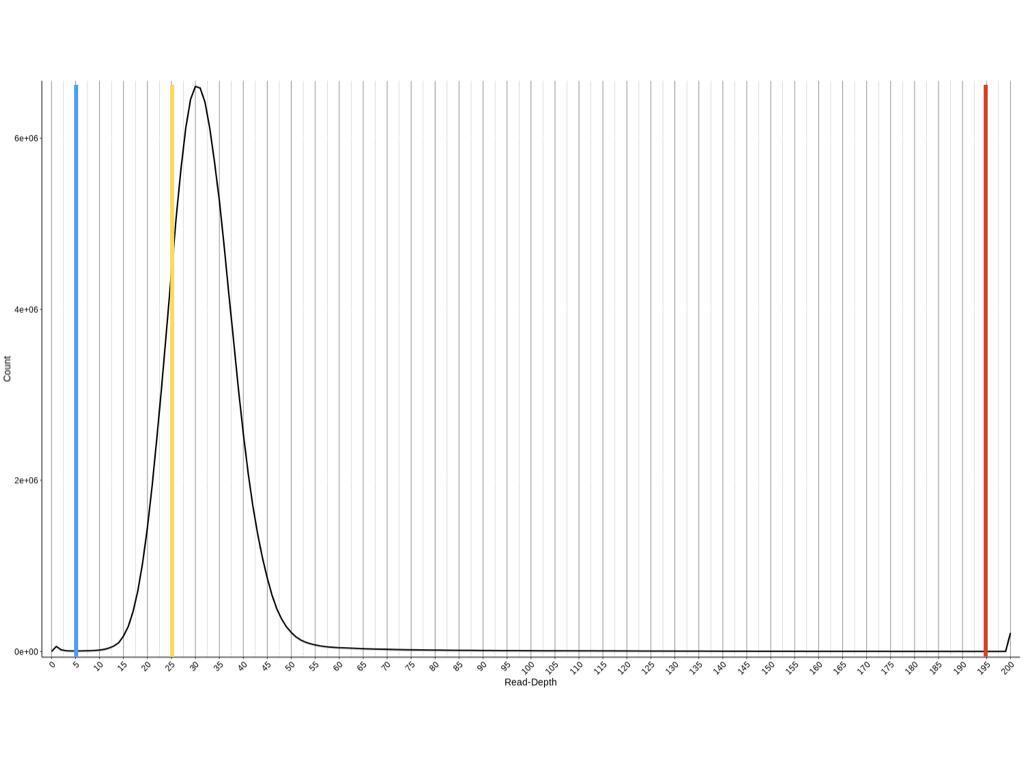


WENGAN-M


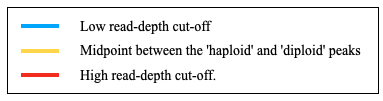


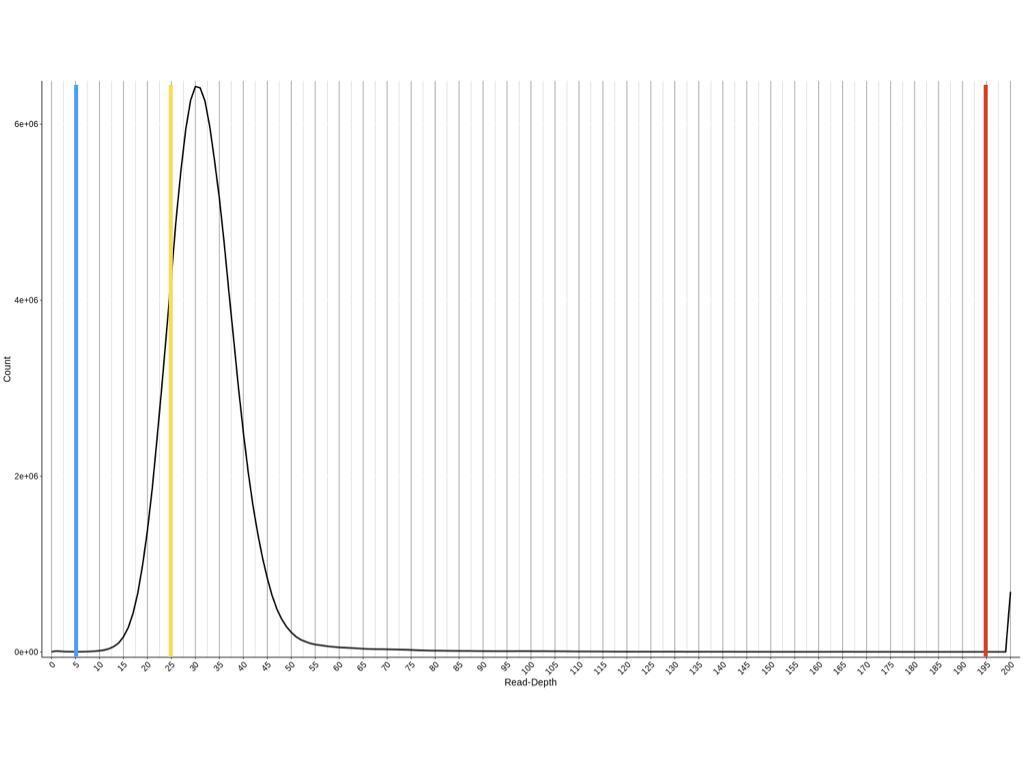


Redbean


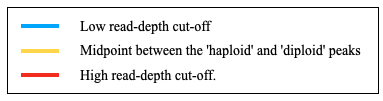


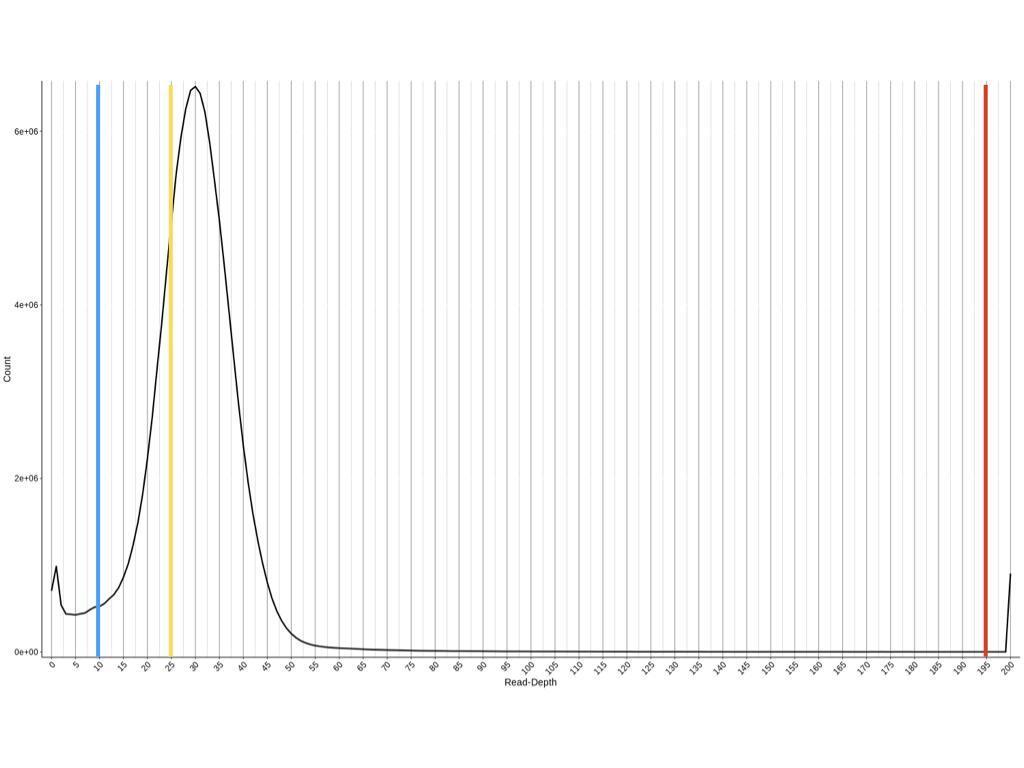


miniasm


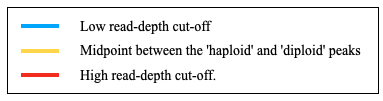


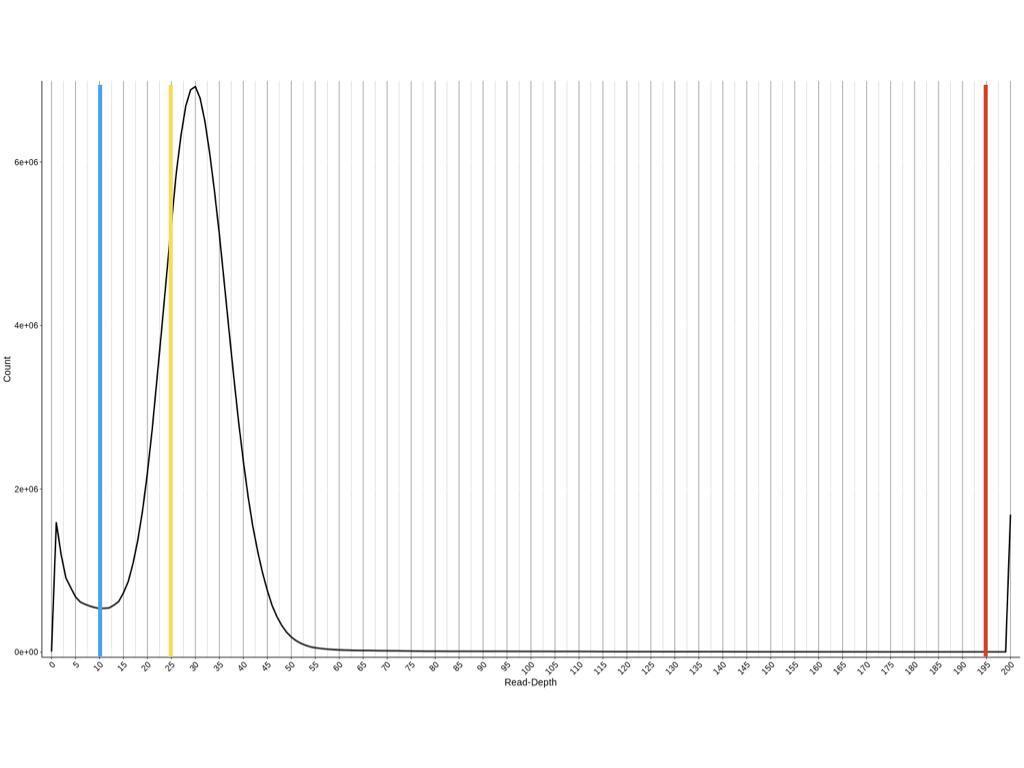


Flye


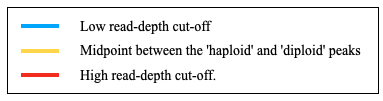


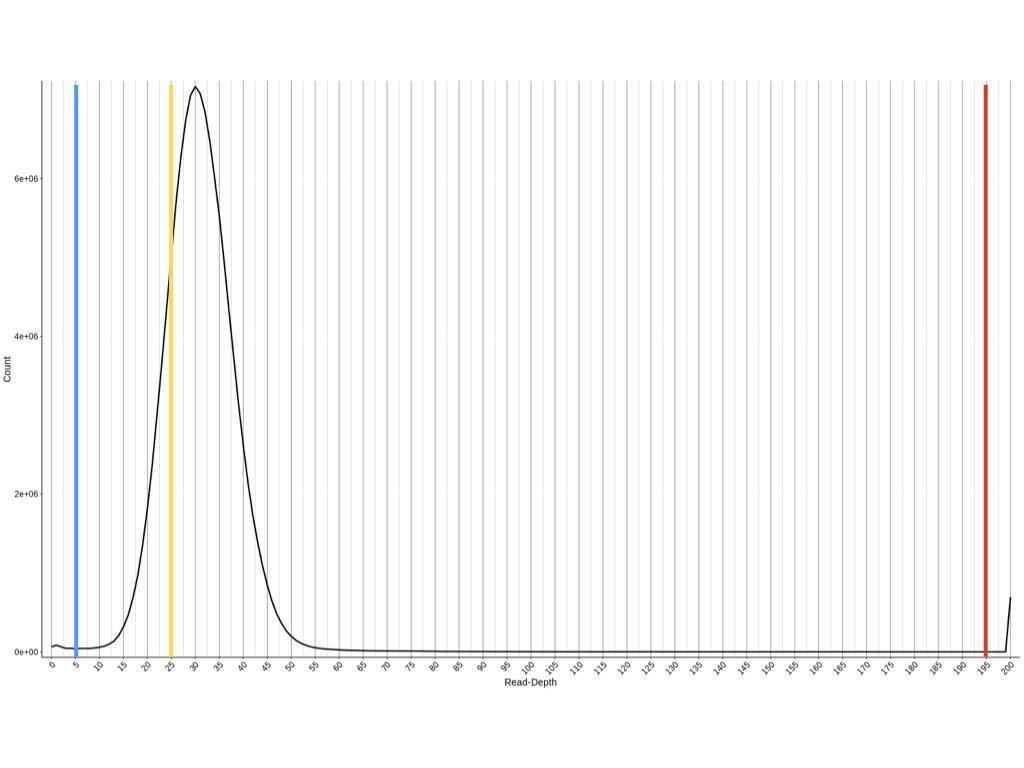


NextDenovo


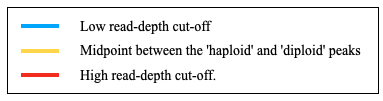


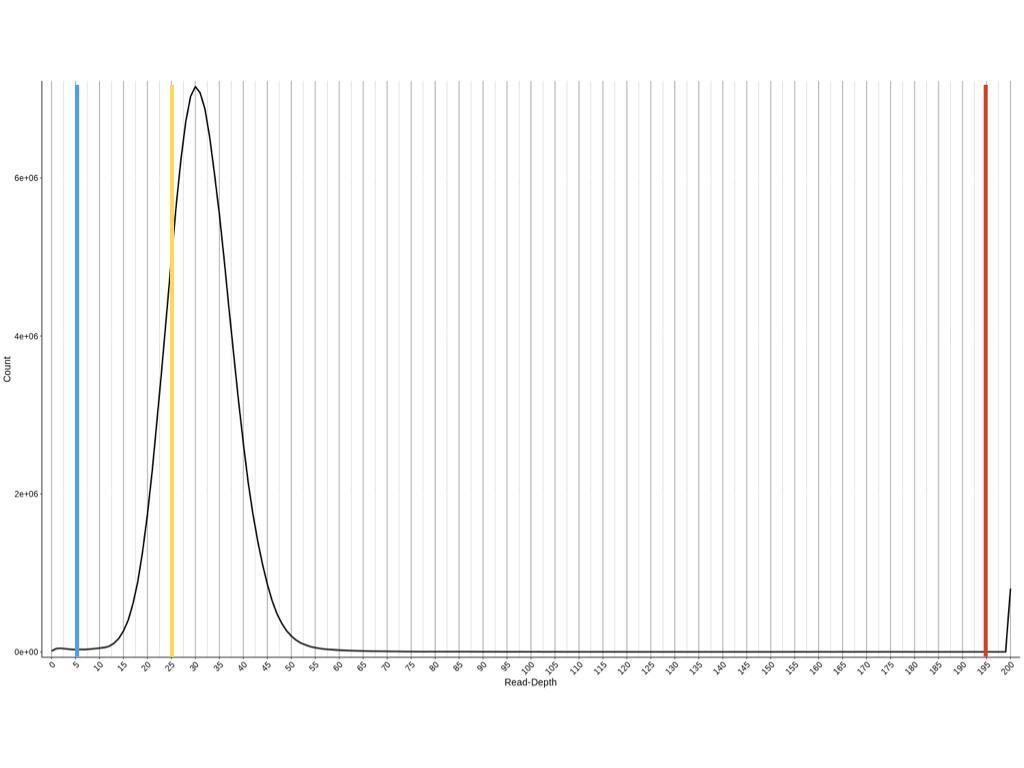


SPAdes


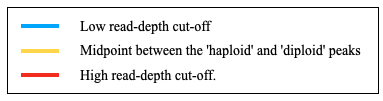


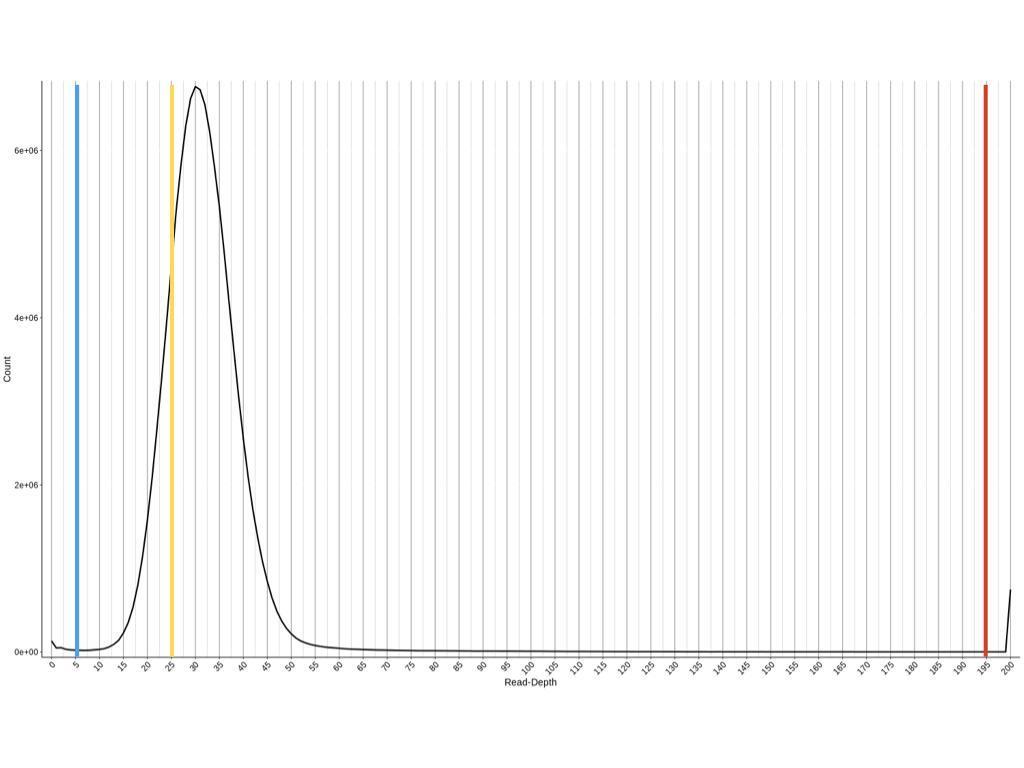


Platanus-allee


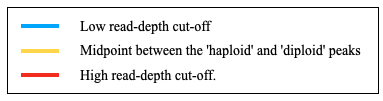


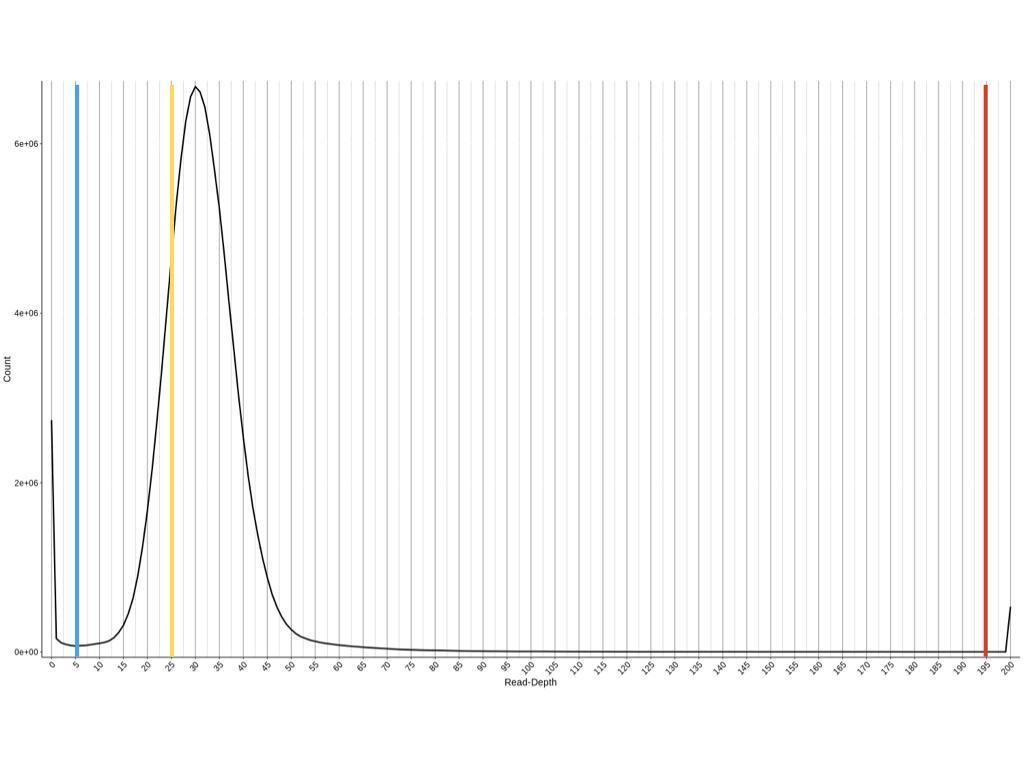


Canu


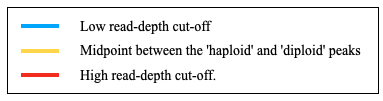


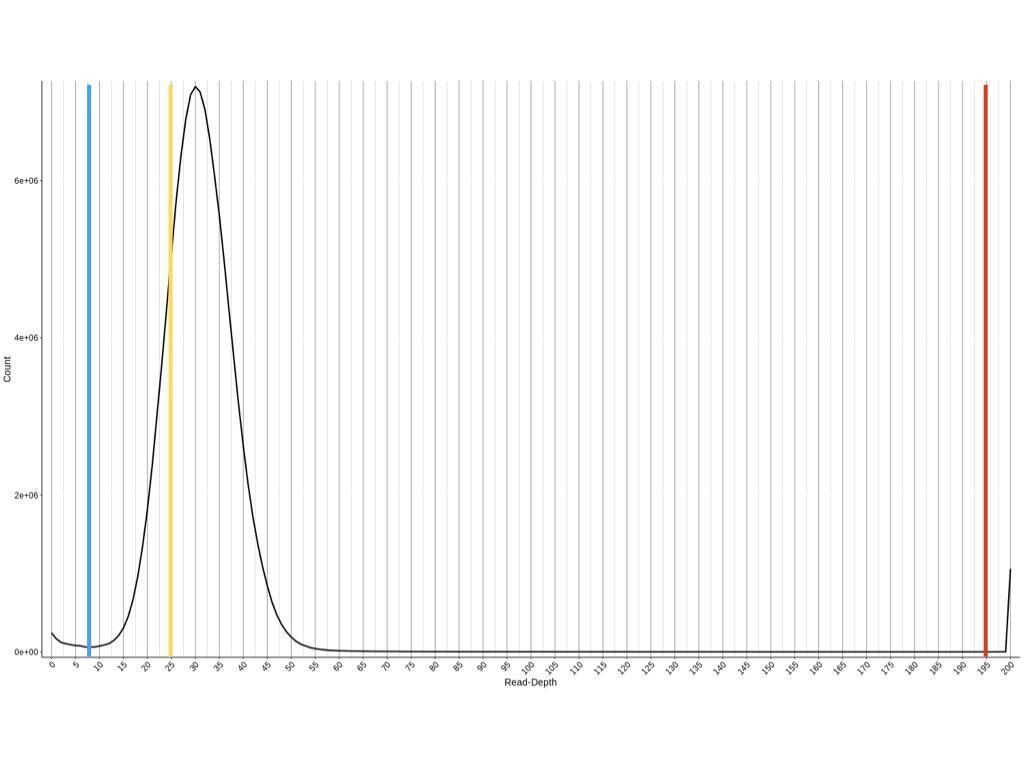


MaSuRCA-F


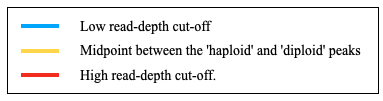


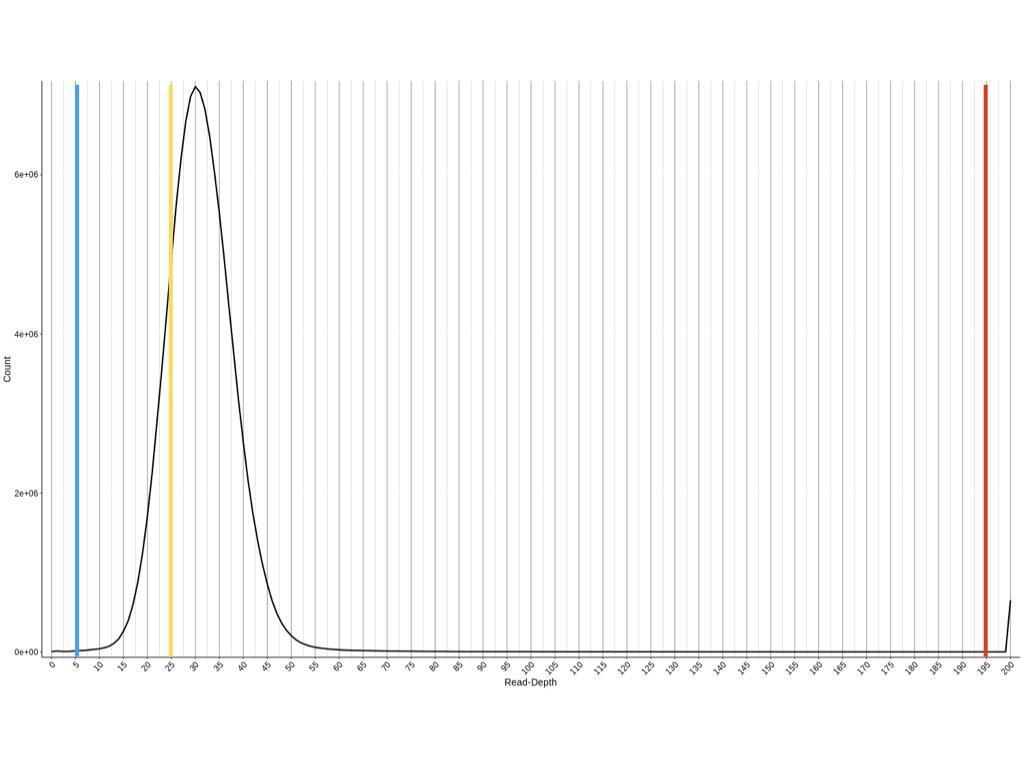


MaSuRCA-C


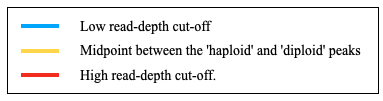


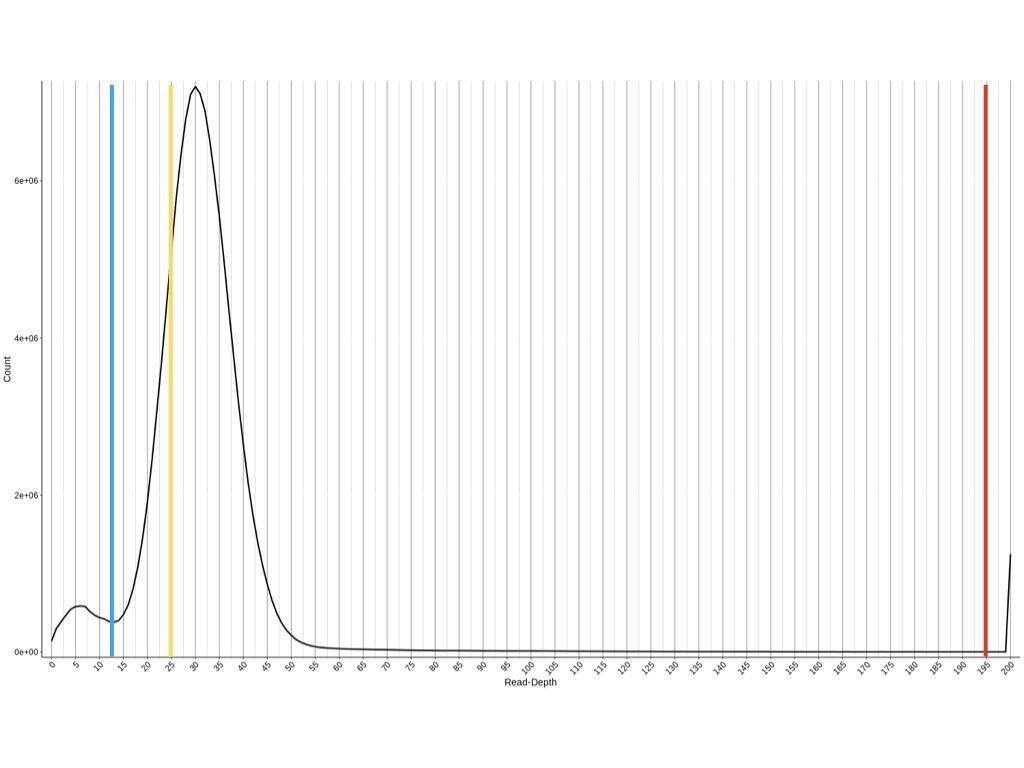


1. *N. putrida* NIES-4239

HASLR


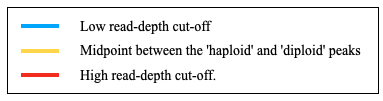


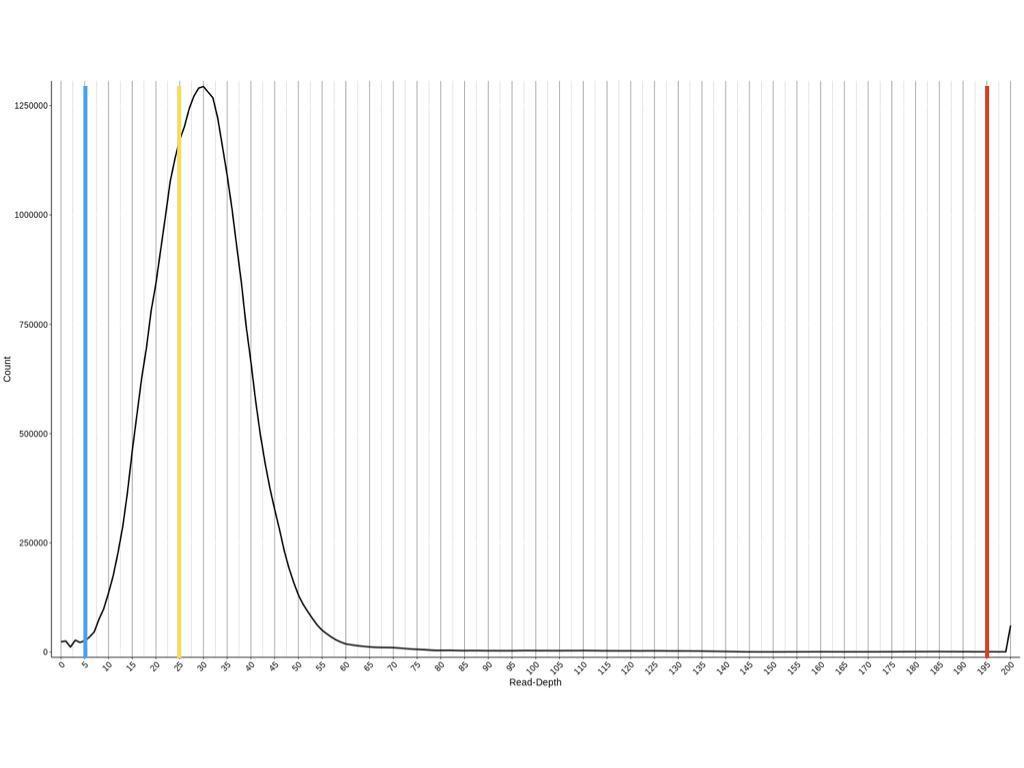


WENGAN-M


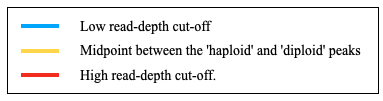


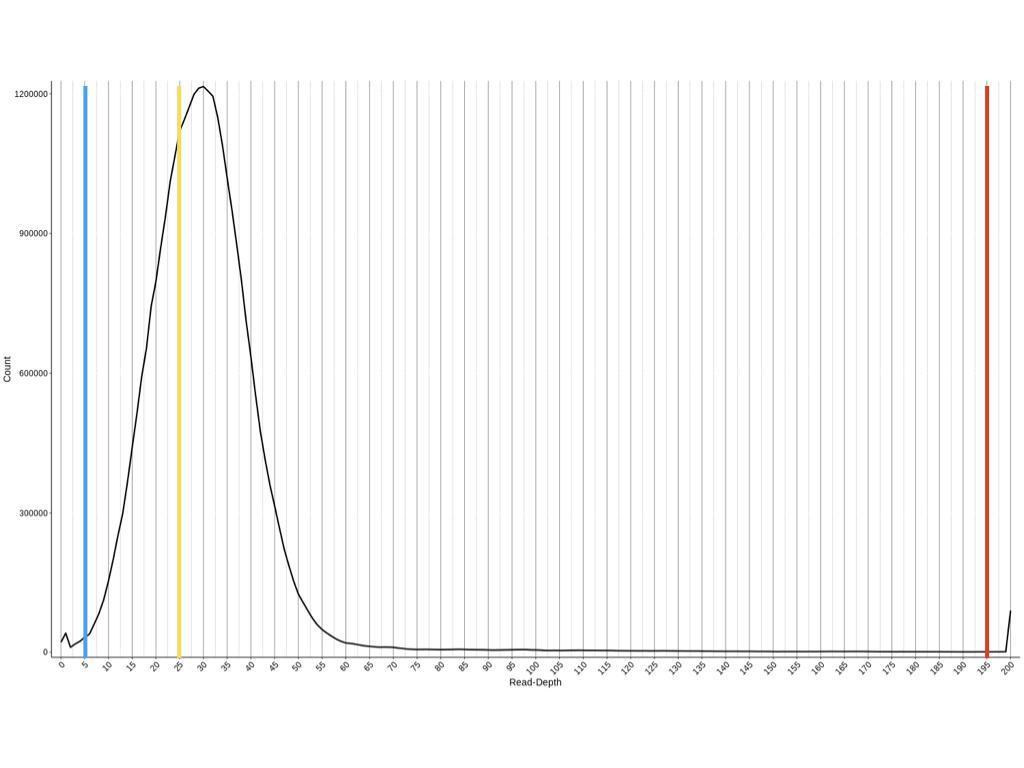


Redbean


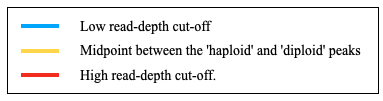


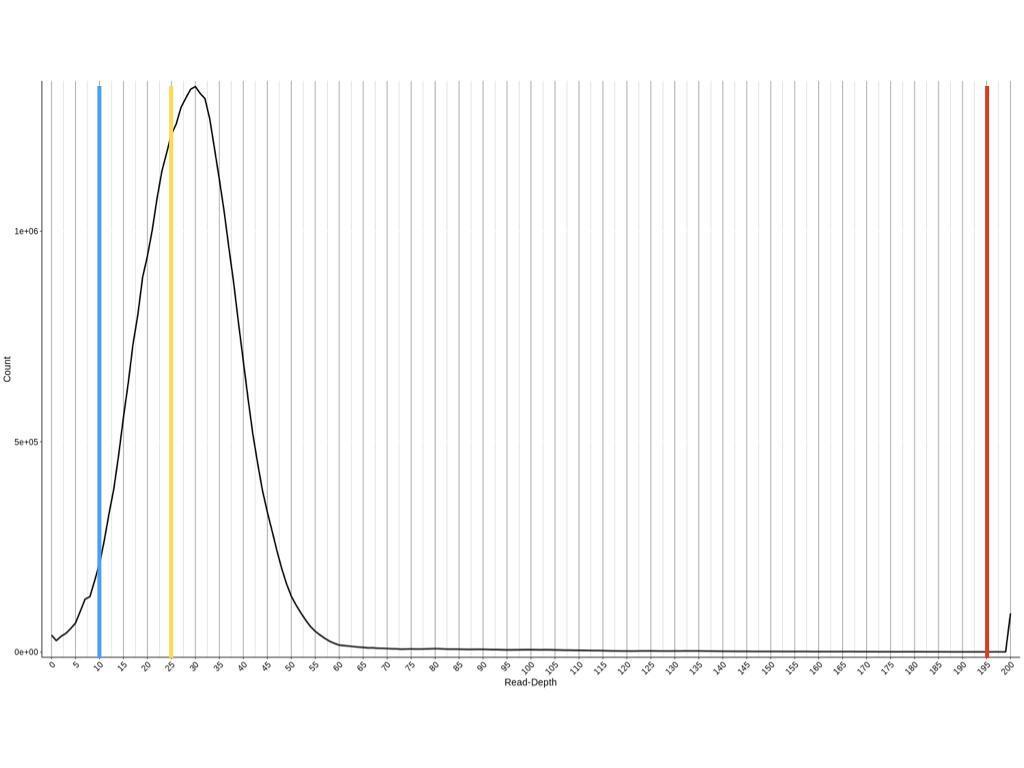


miniasm


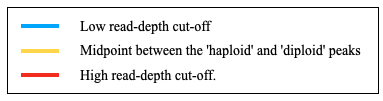


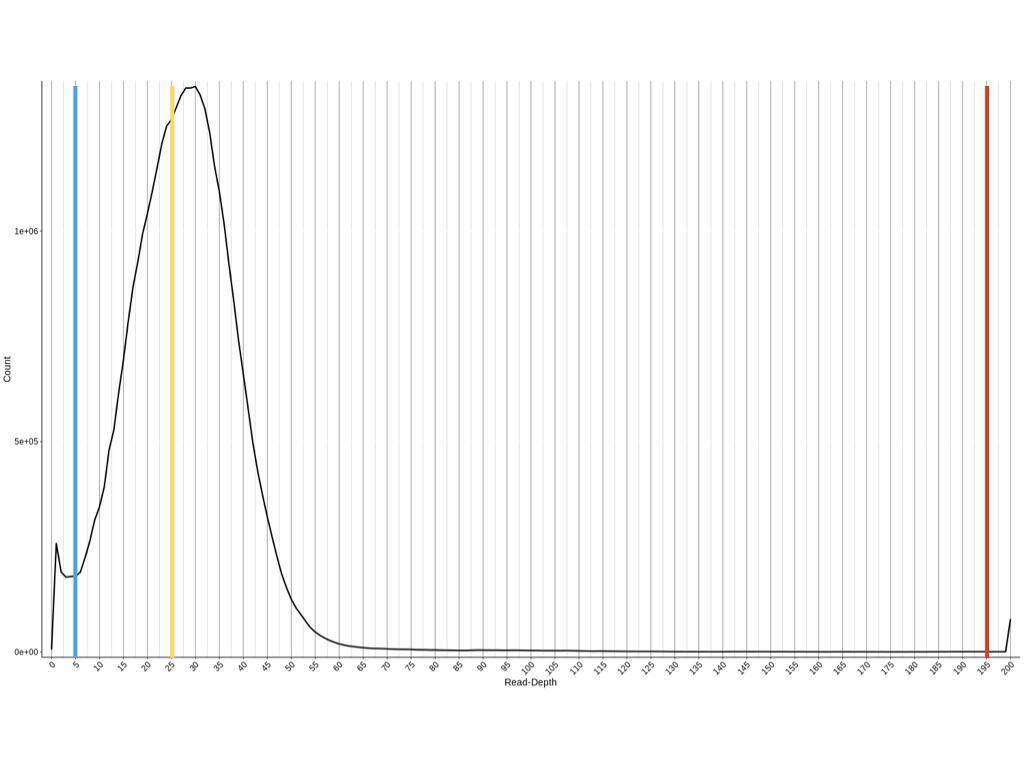


Flye


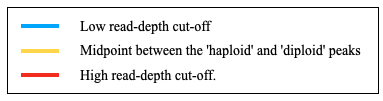


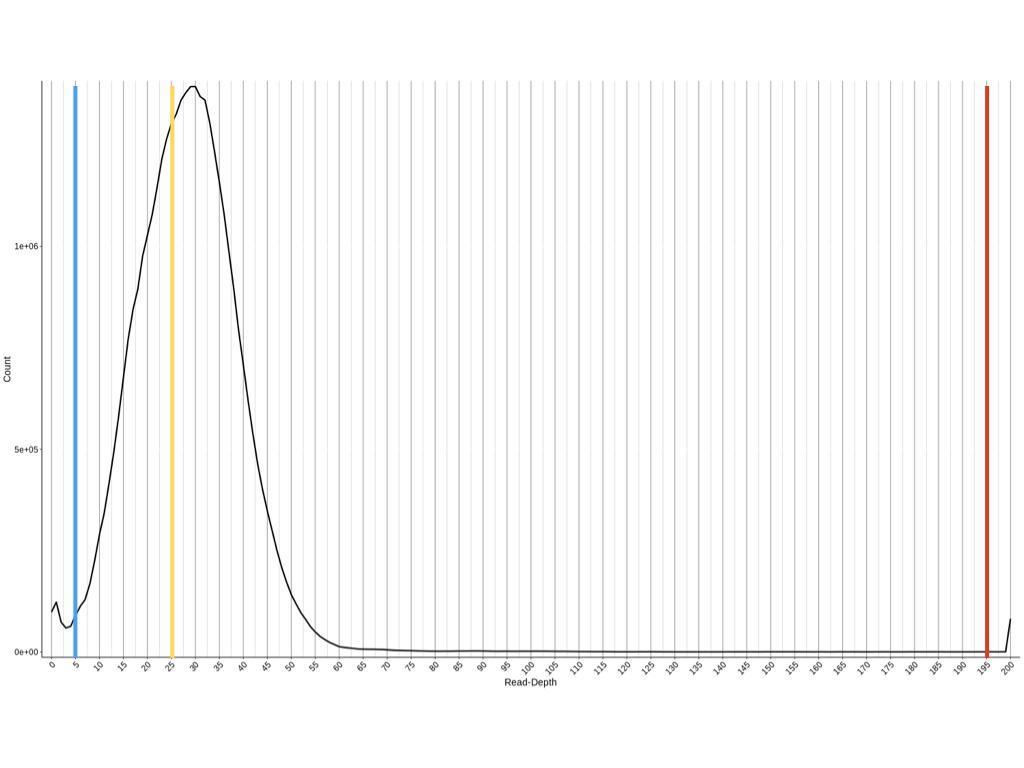


NextDenovo


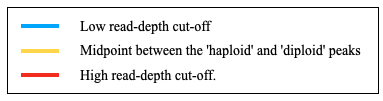


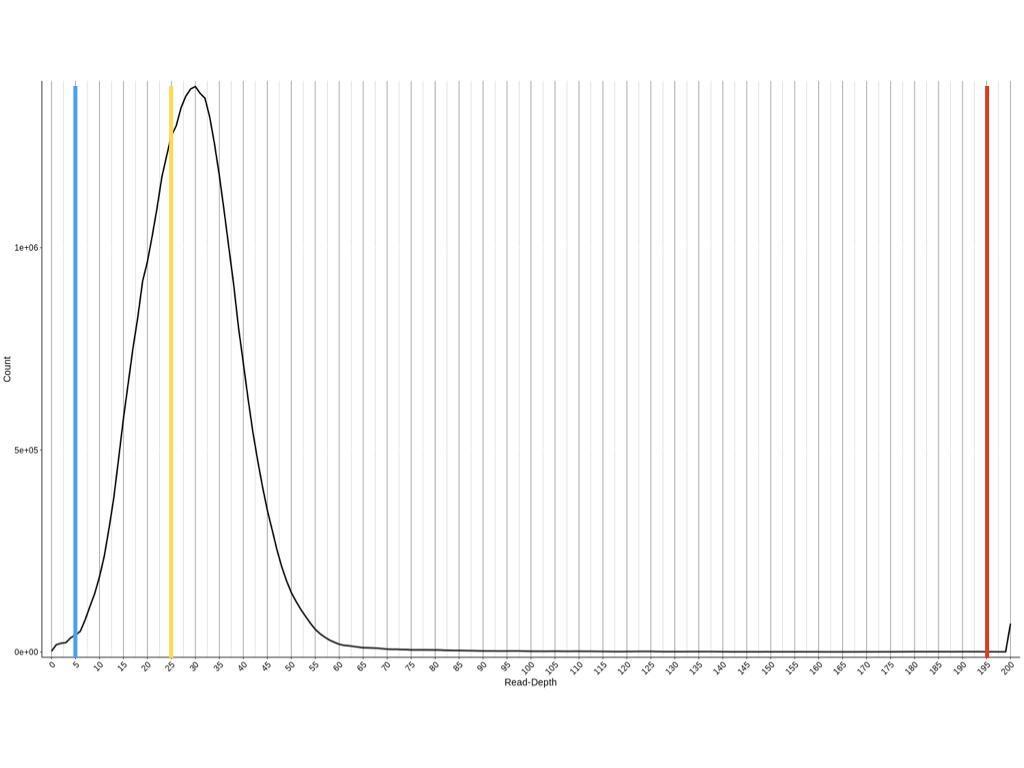


SPAdes


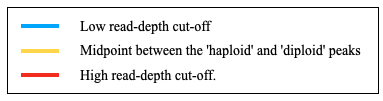


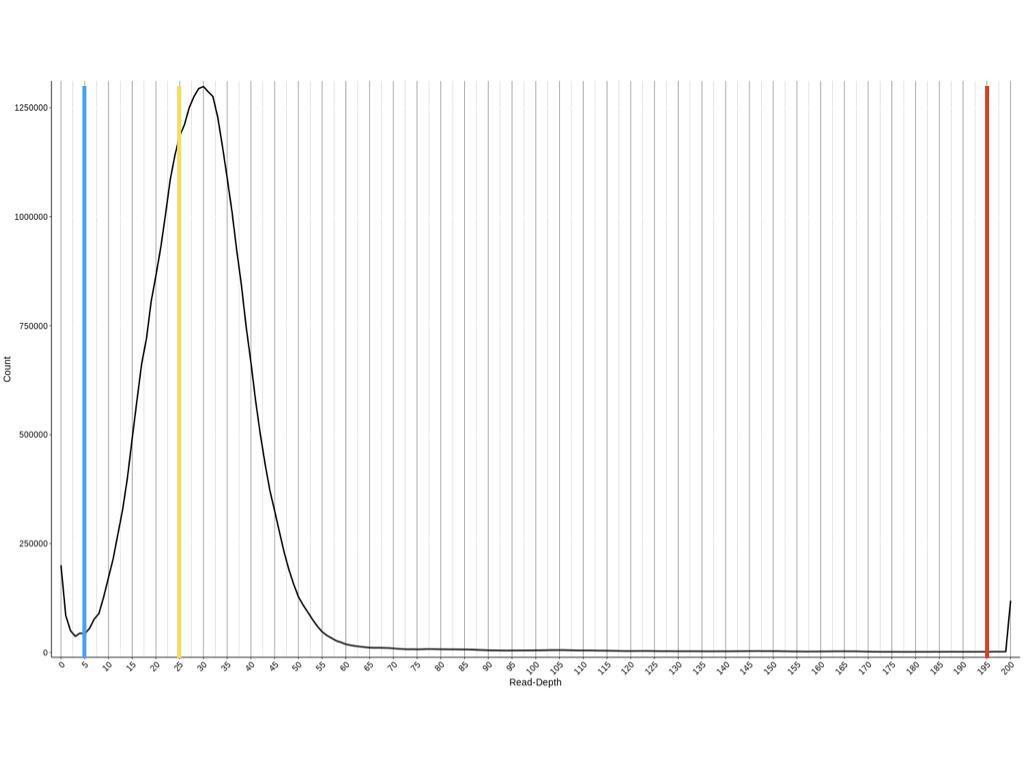


Platanus-allee


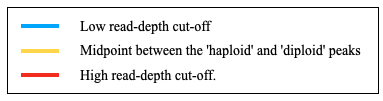


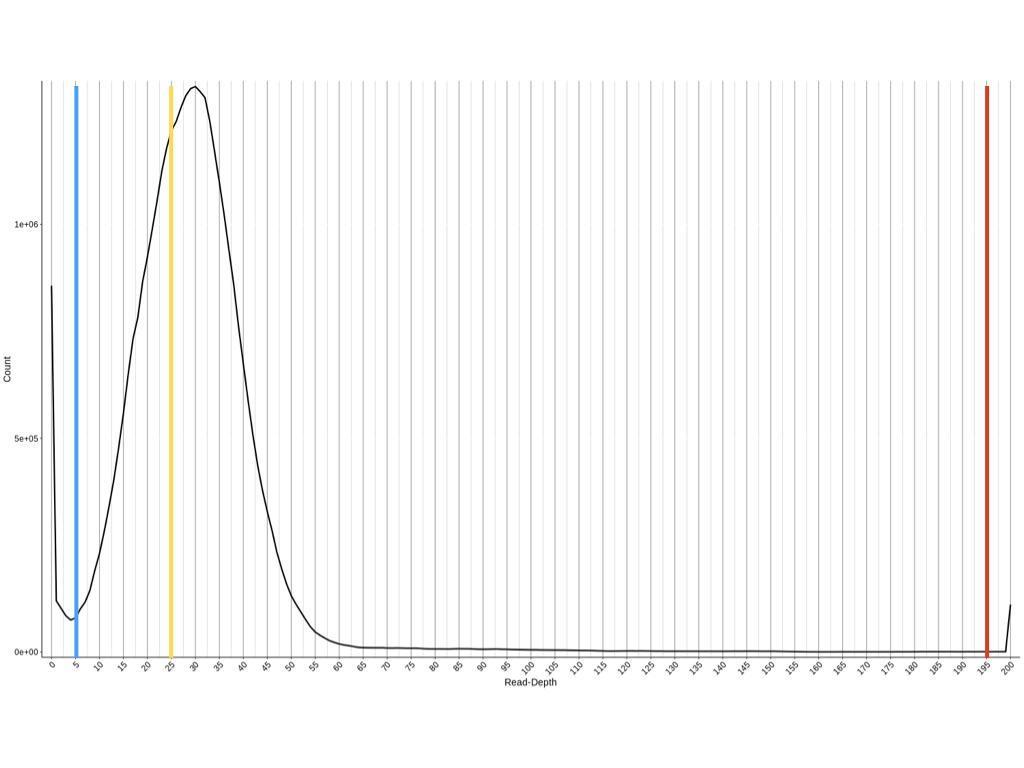


Canu


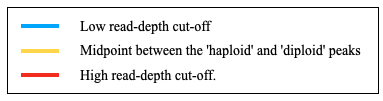


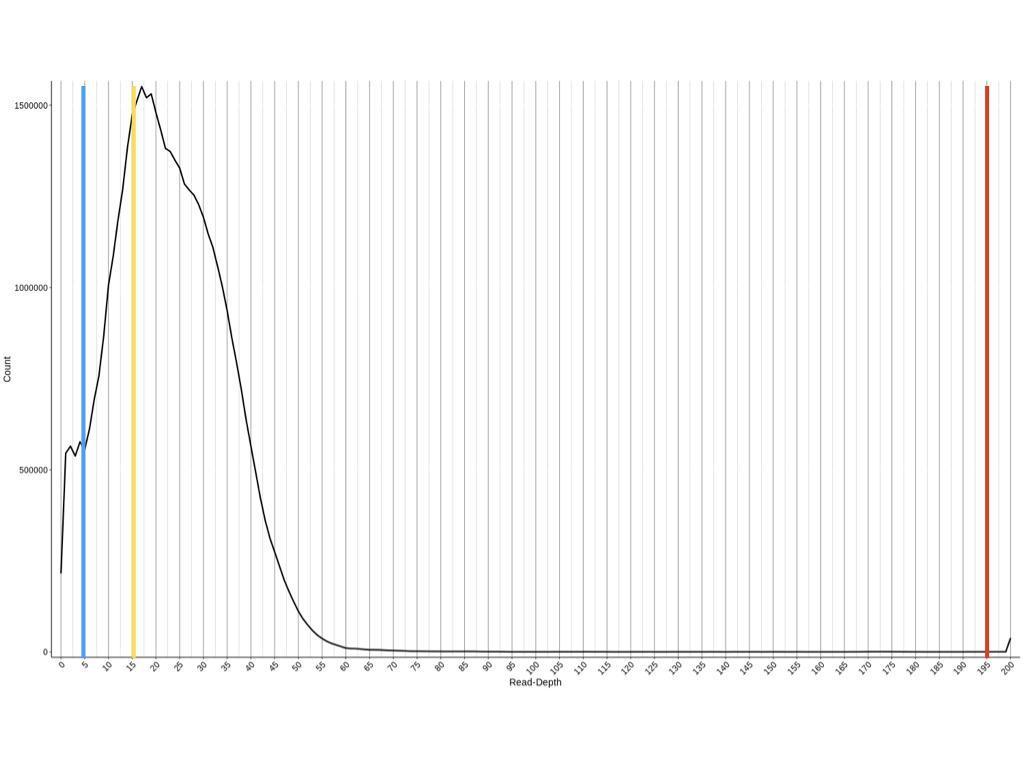


MaSuRCA-F


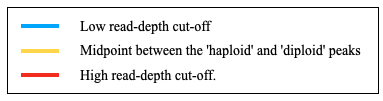


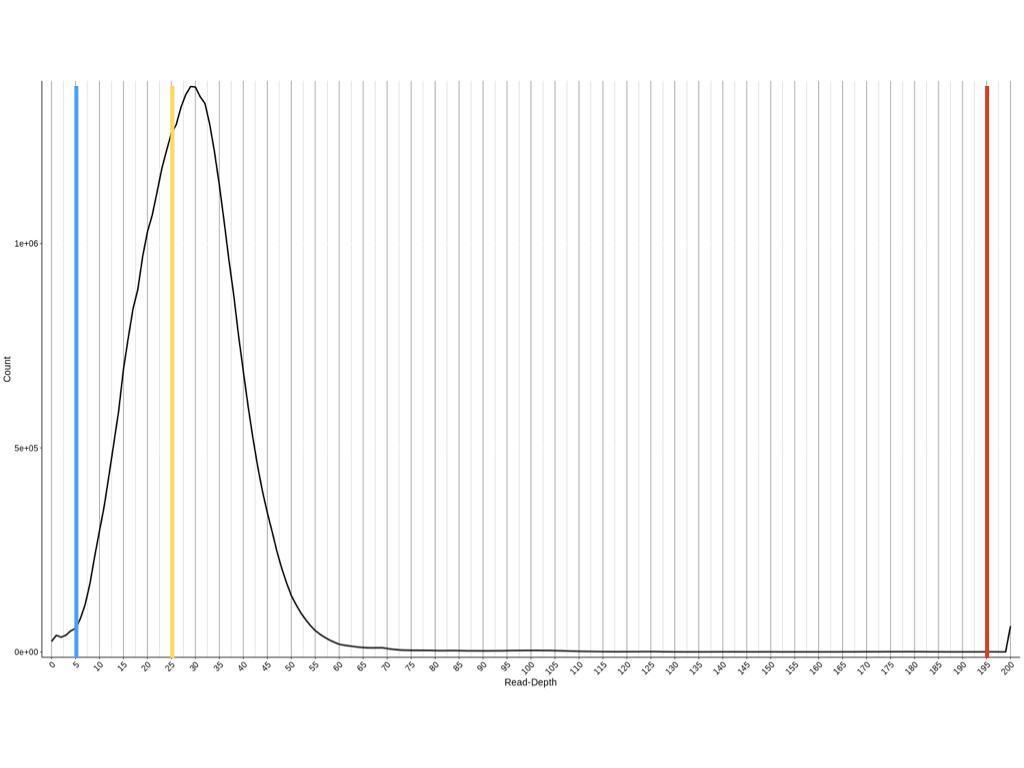


MaSuRCA-C


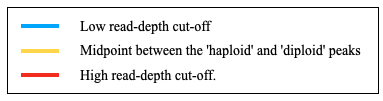


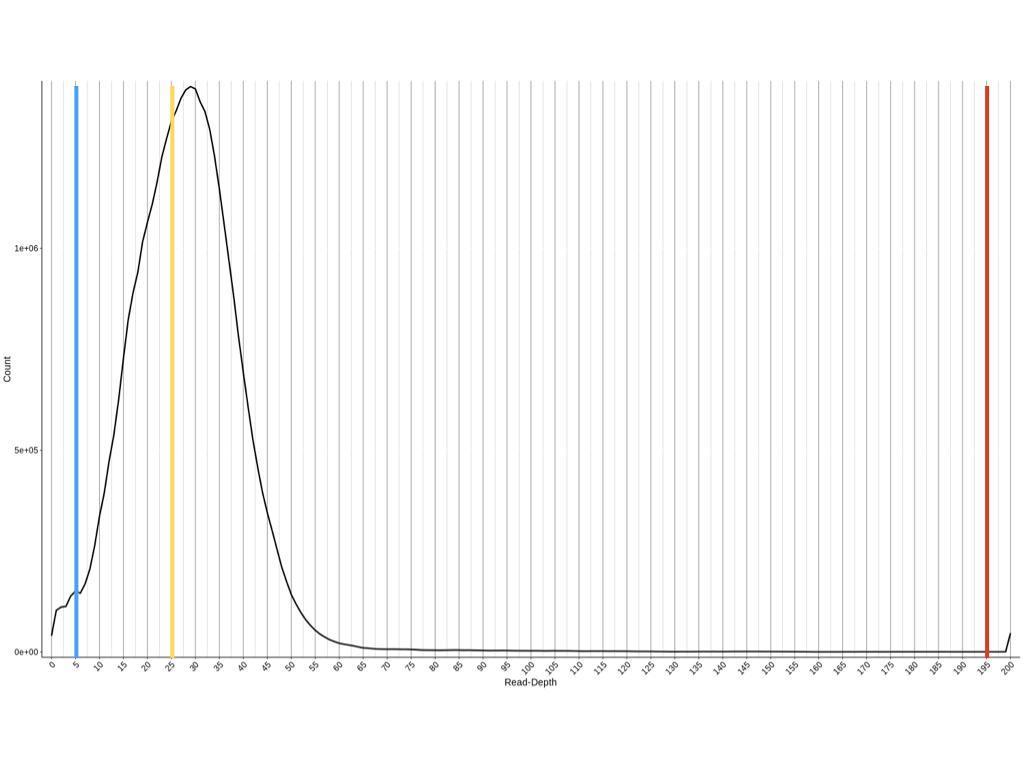


1. *L. calcarifer*

HASLR


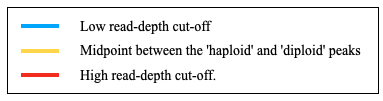


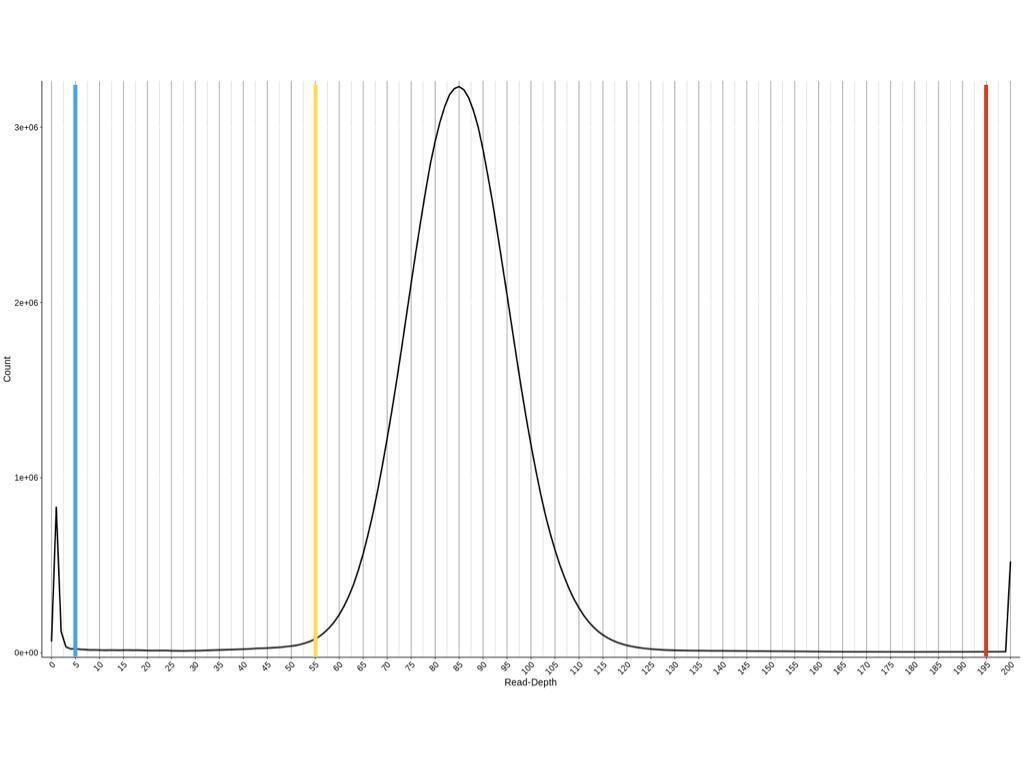


WENGAN-M


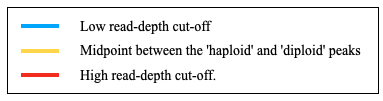


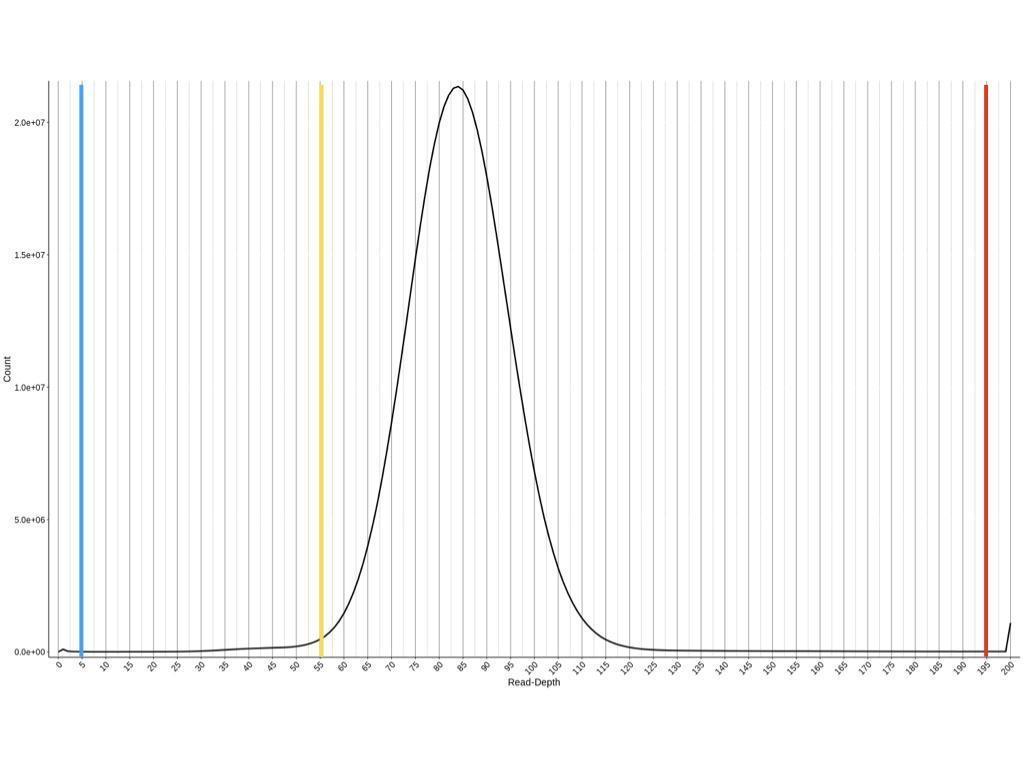


Redbean


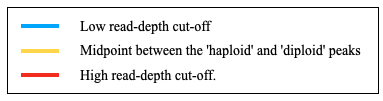


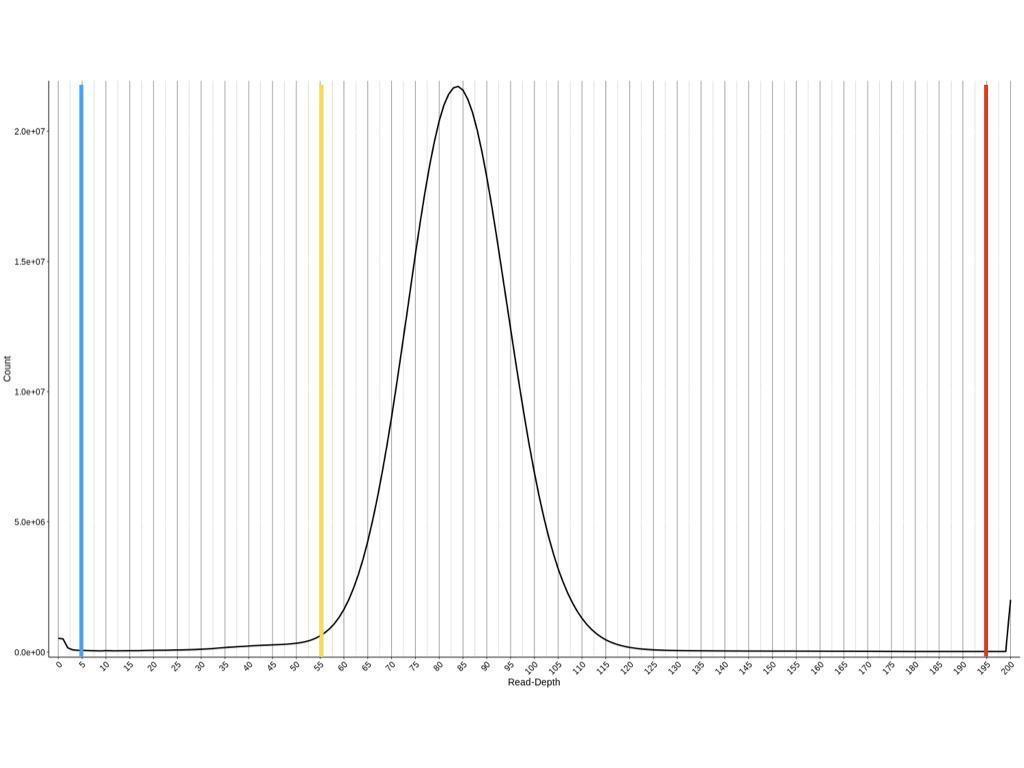


miniasm


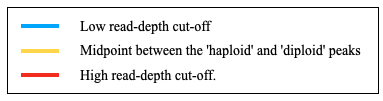


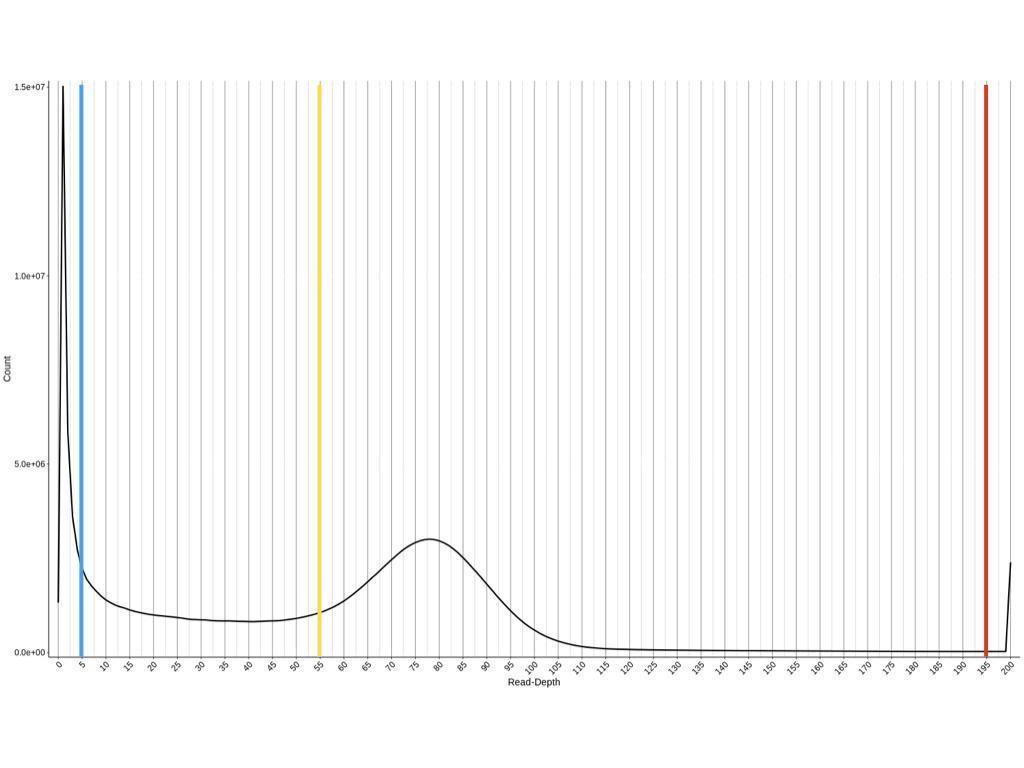


Flye


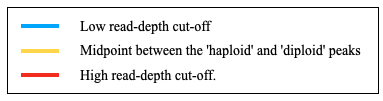


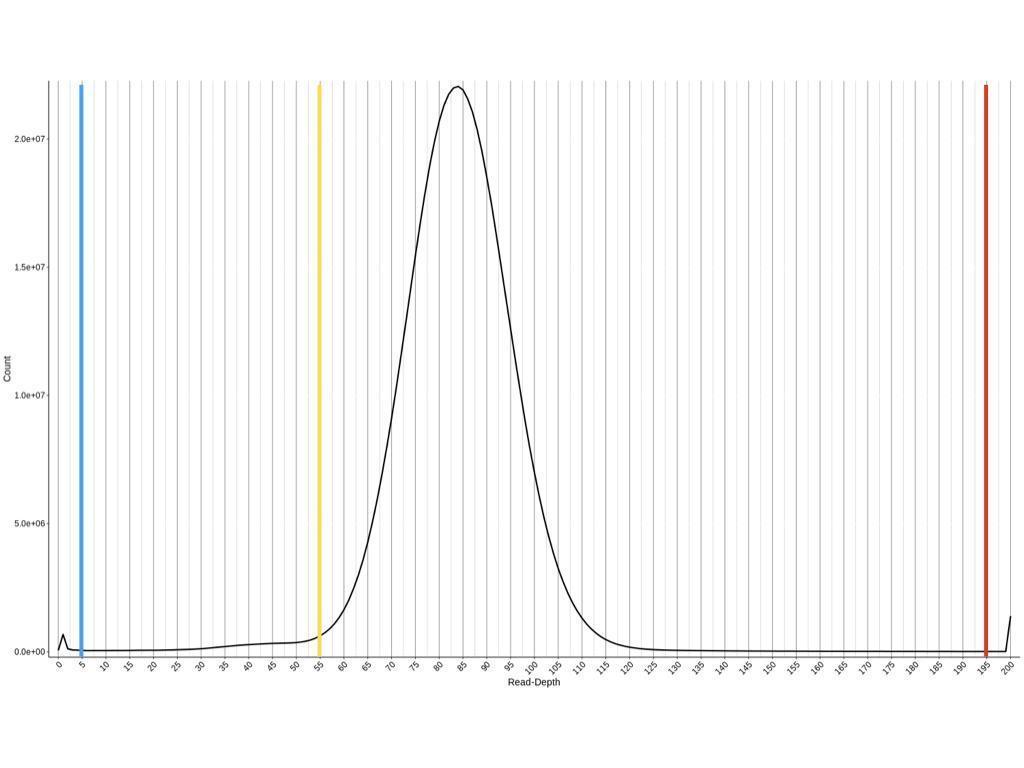


NextDenovo


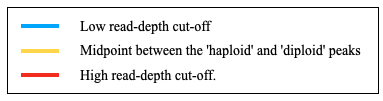


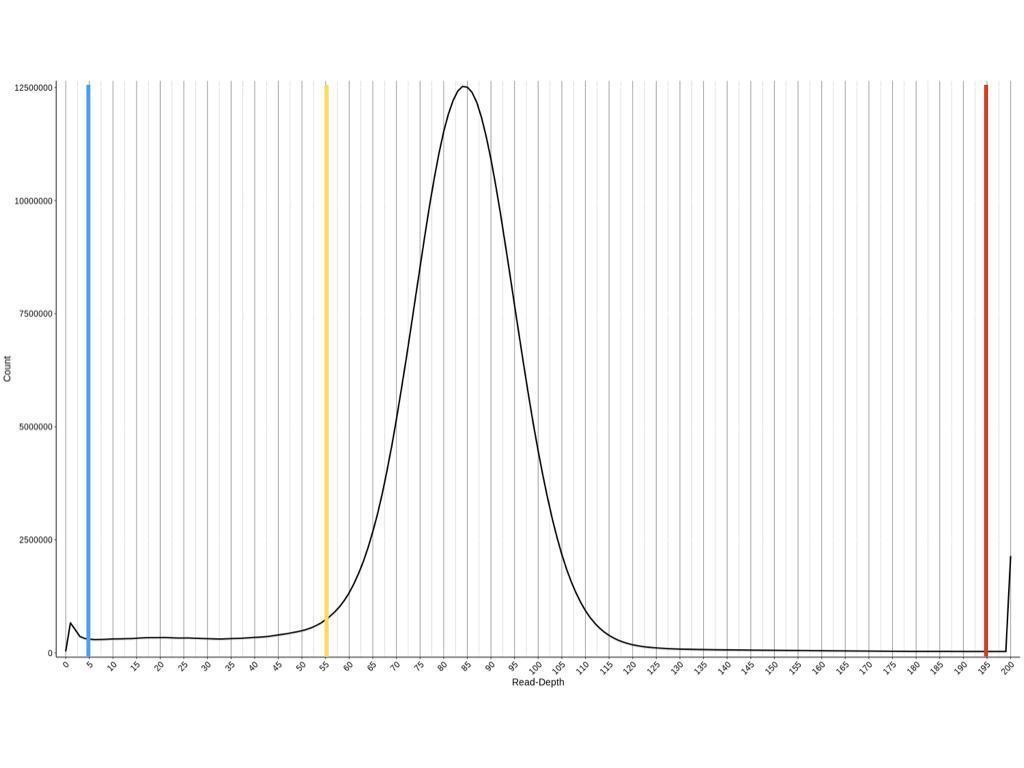


SPAdes


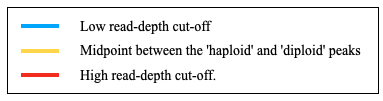


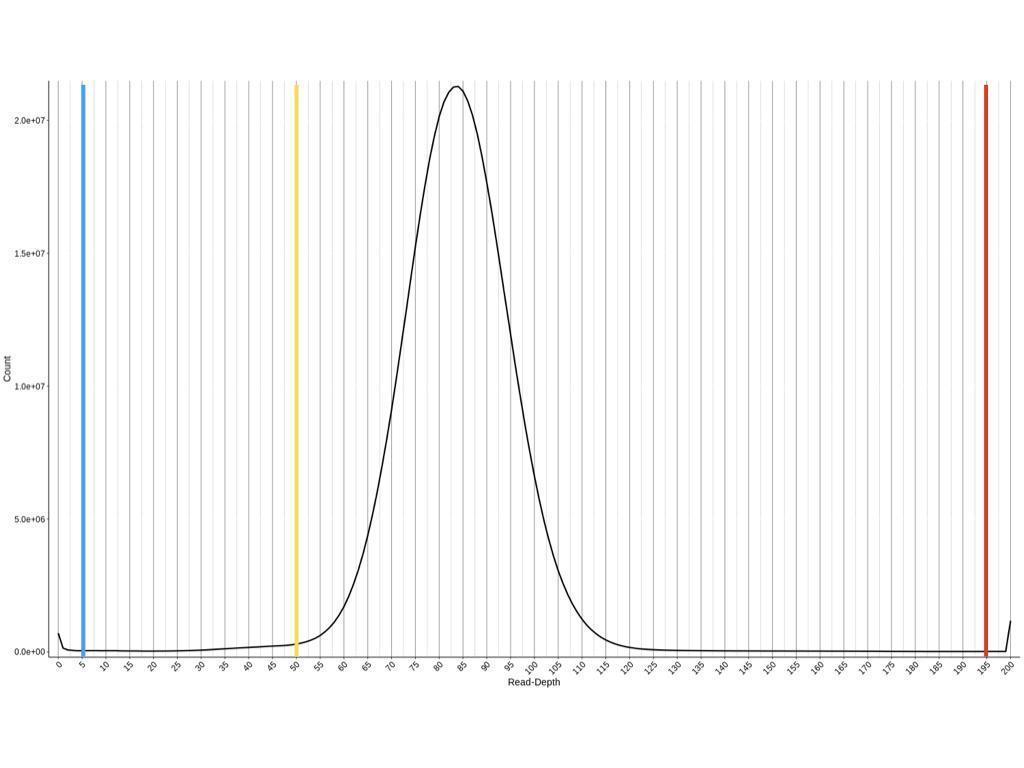


Platanus-allee


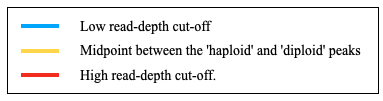


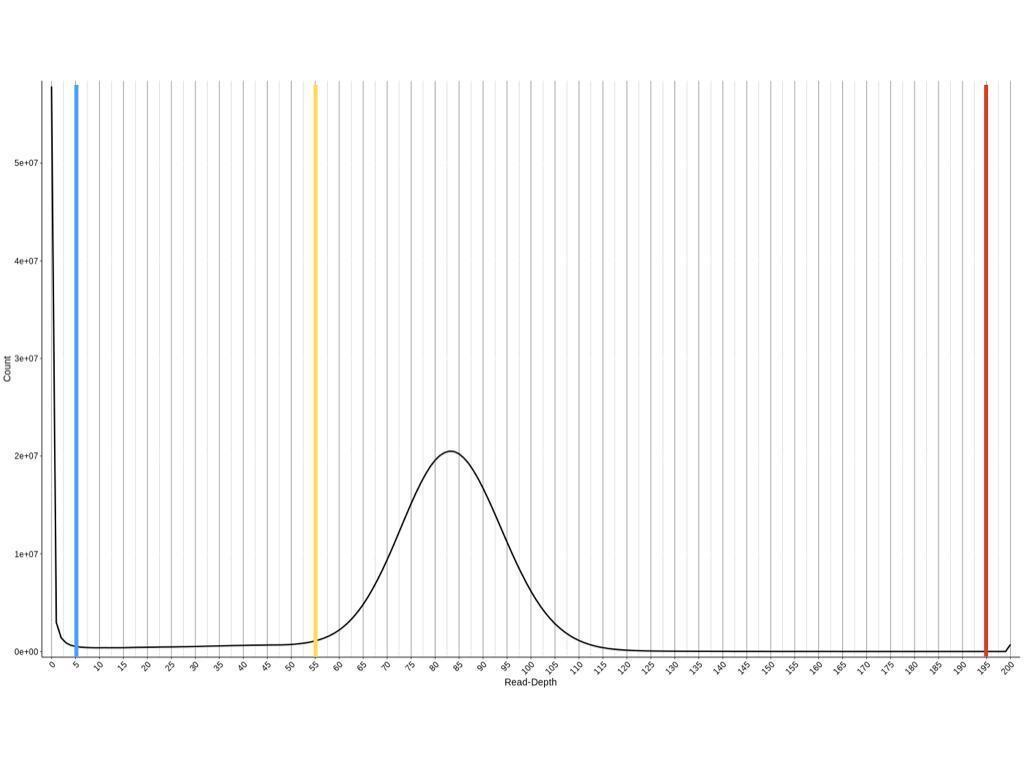


Canu

Timeout

MaSuRCA-F


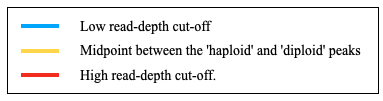


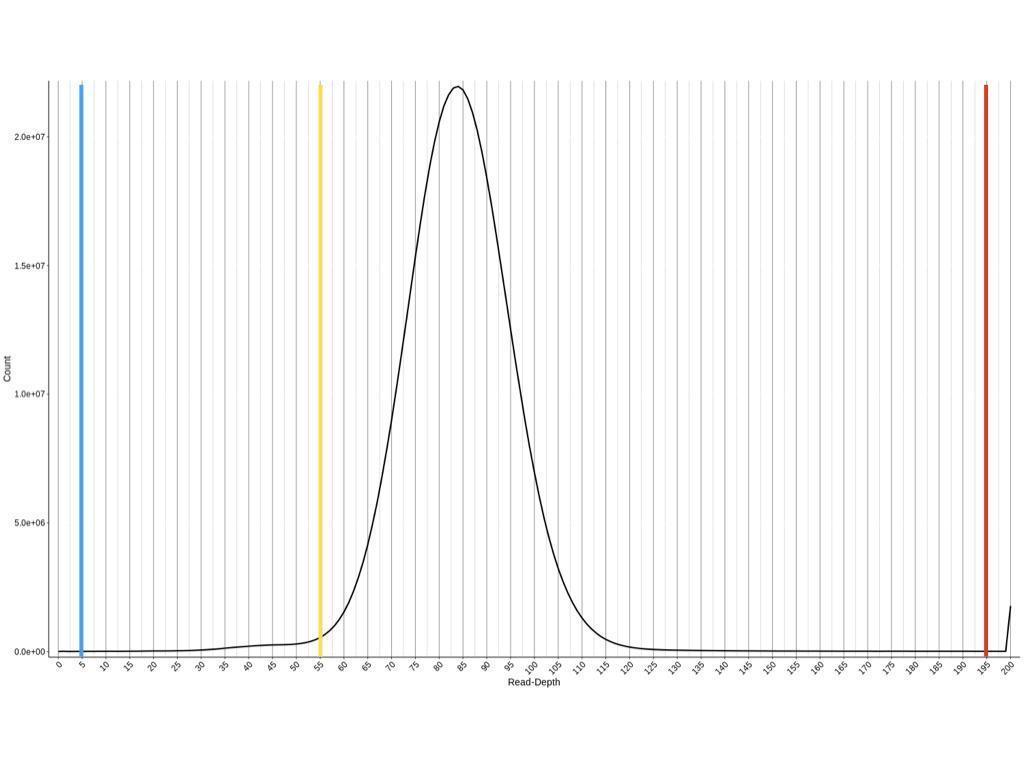


MaSuRCA-C


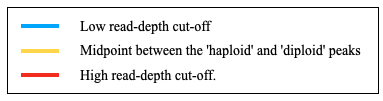


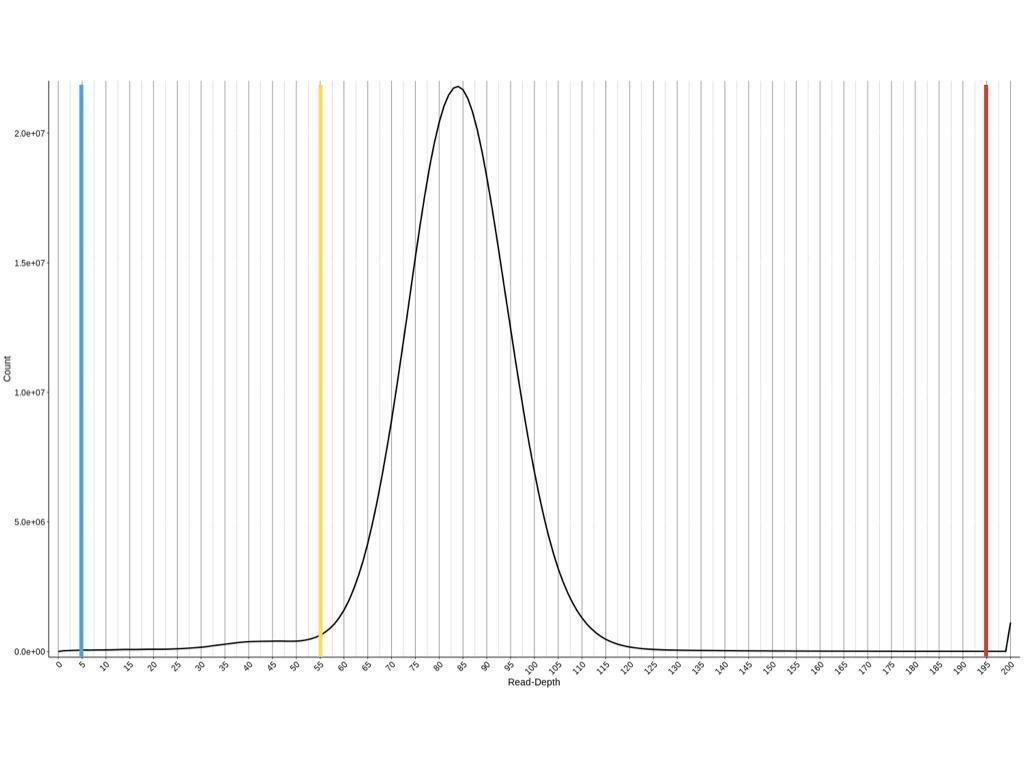


1. *S. sitiens*

HASLR


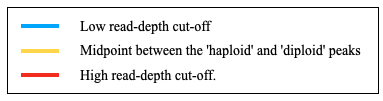


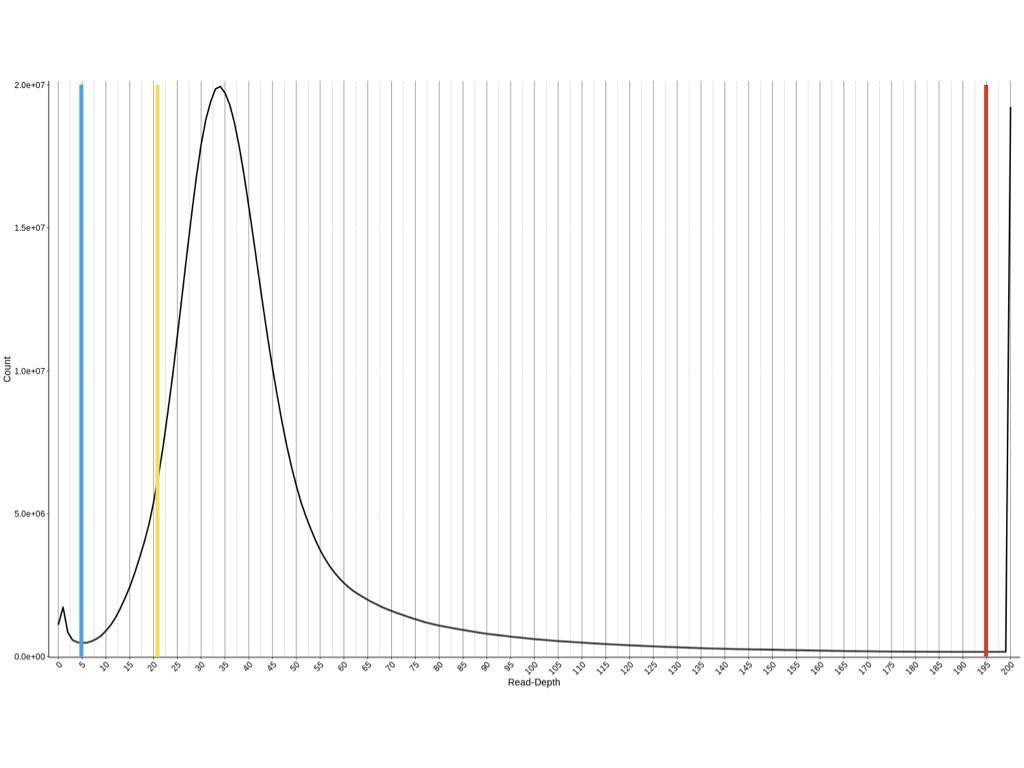


WENGAN-M


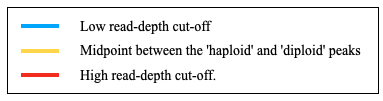


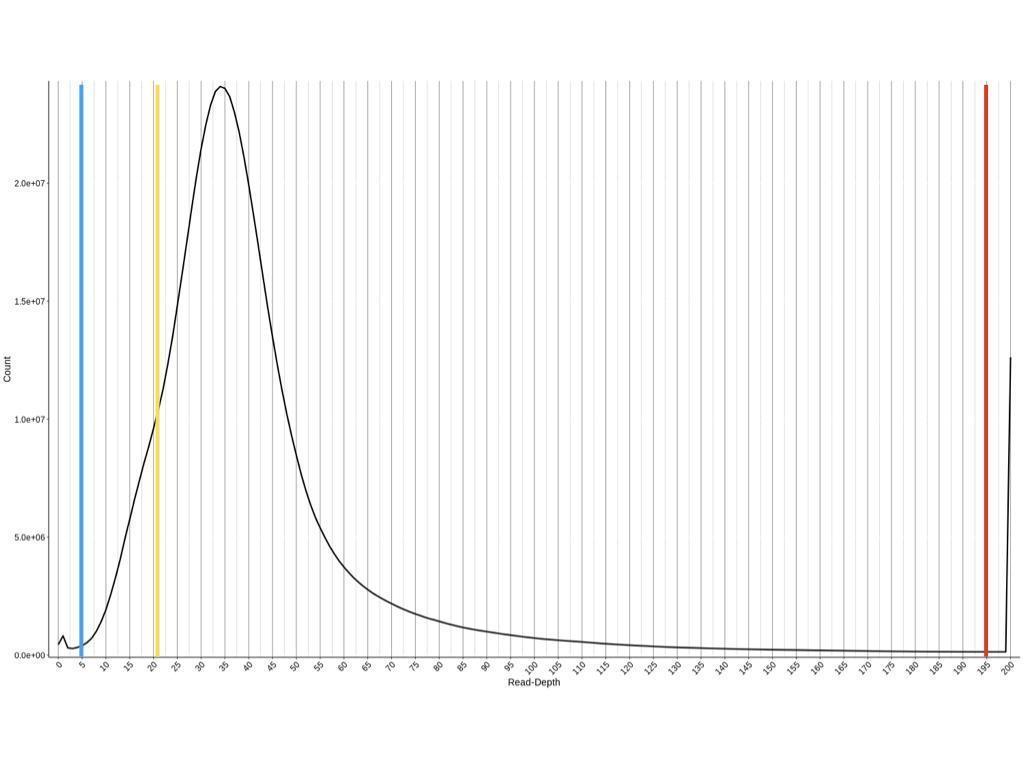


Redbean


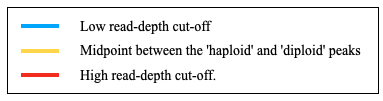


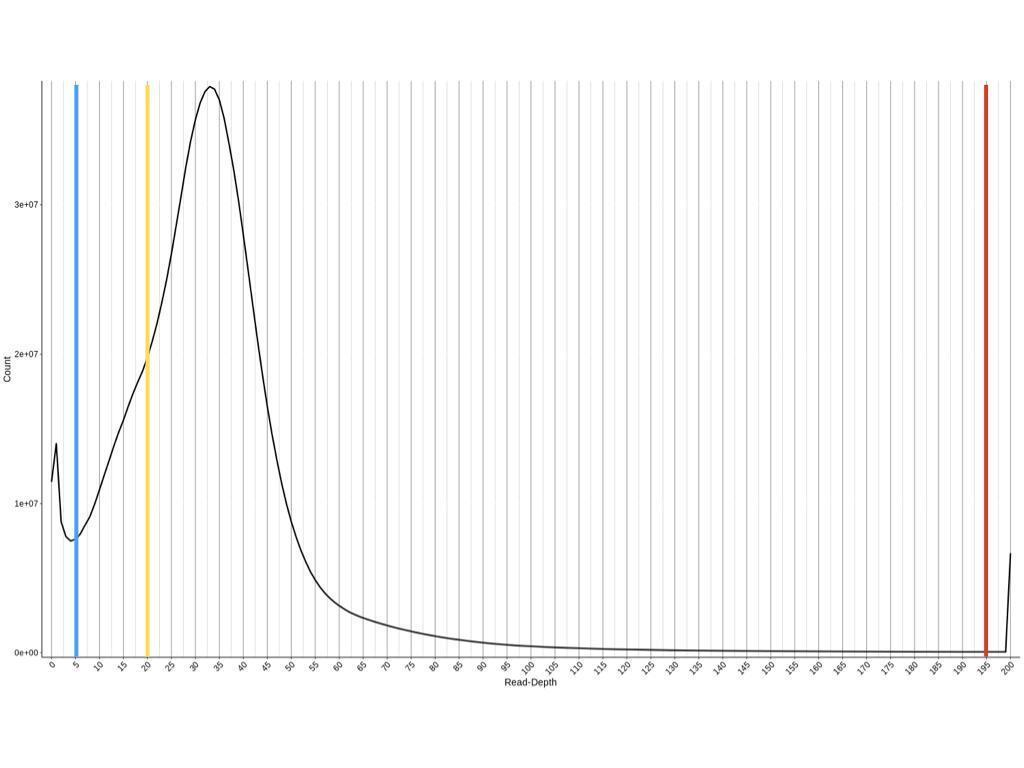


miniasm


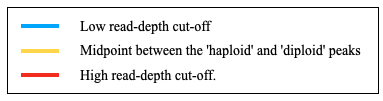


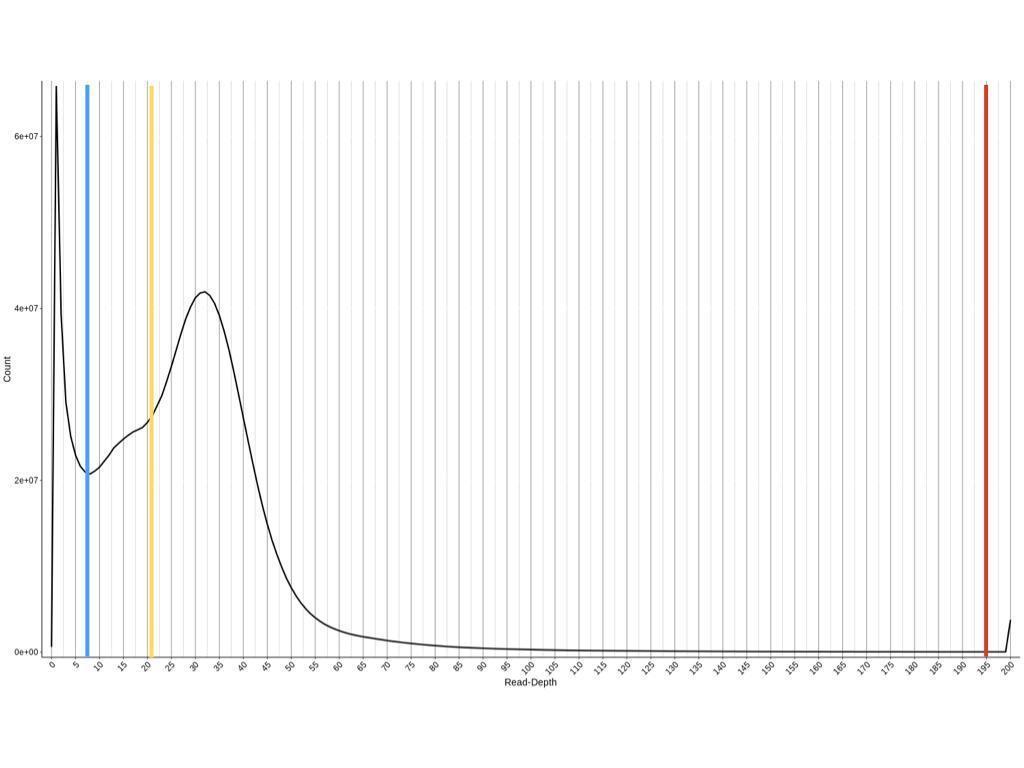


Flye


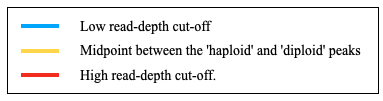


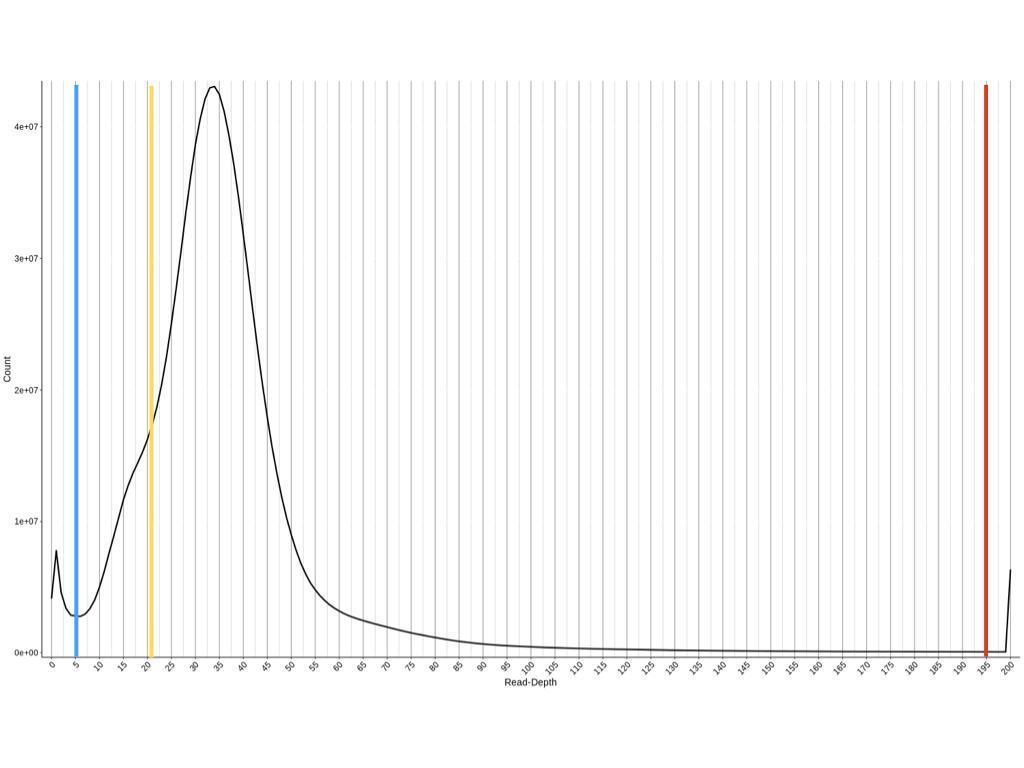


NextDenovo


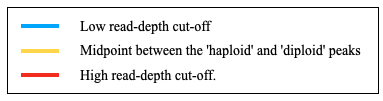


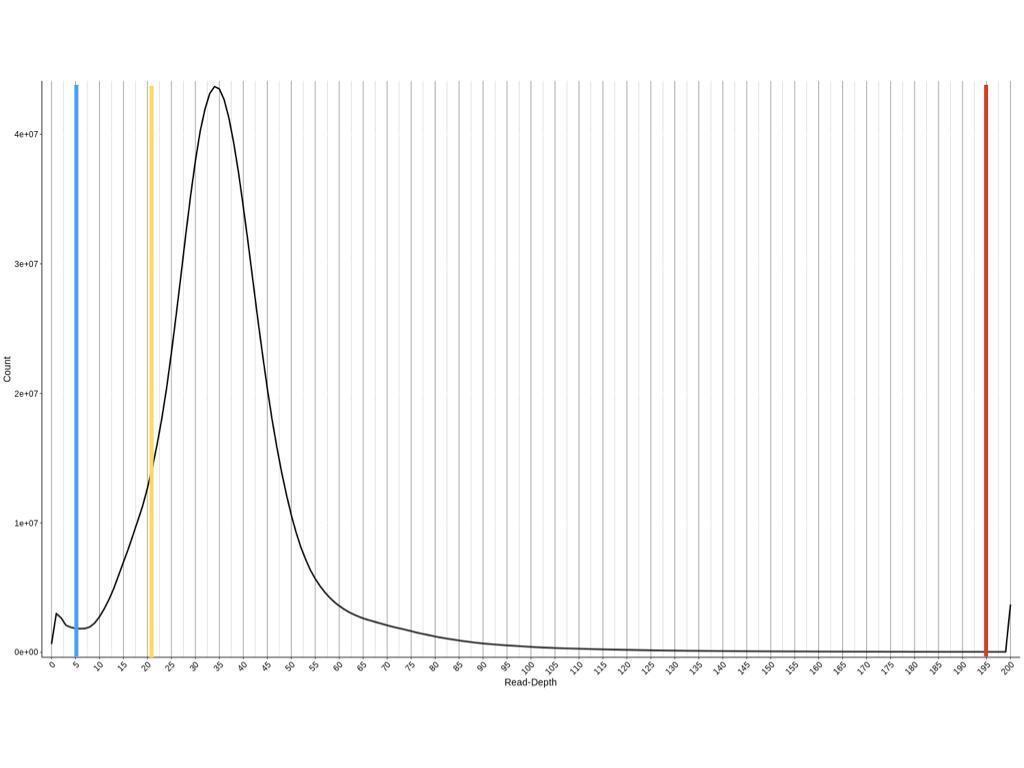


SPAdes


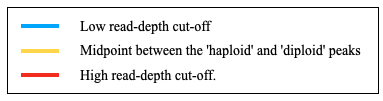


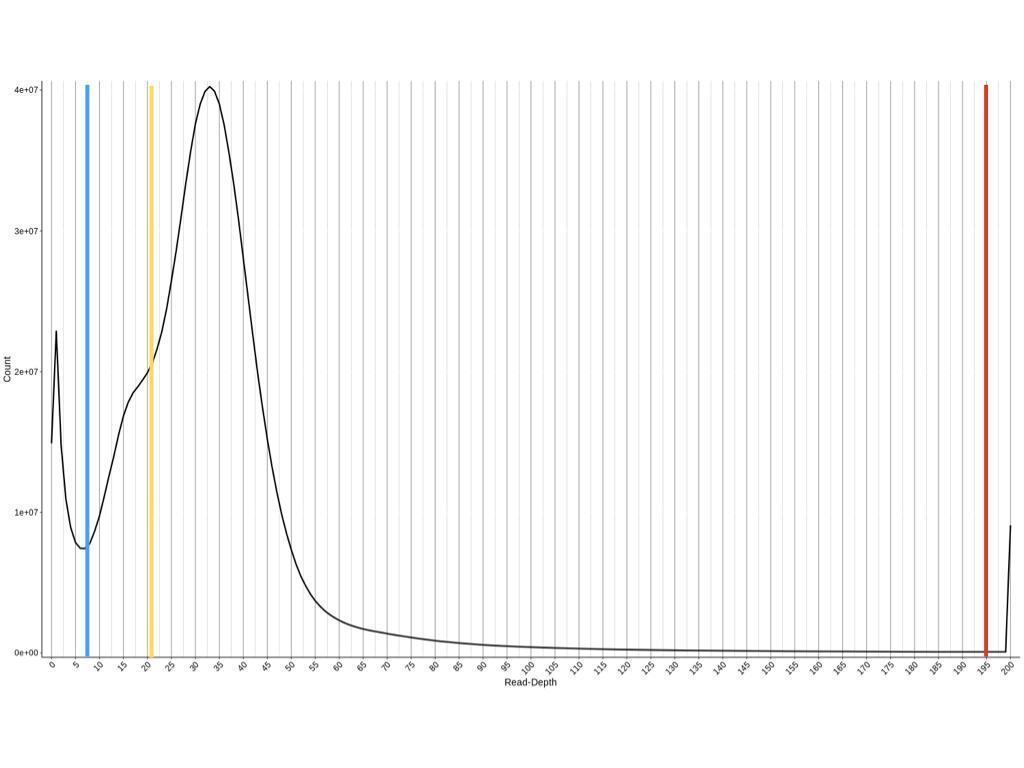


Platanus-allee


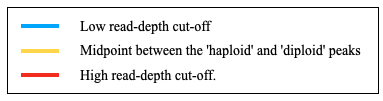


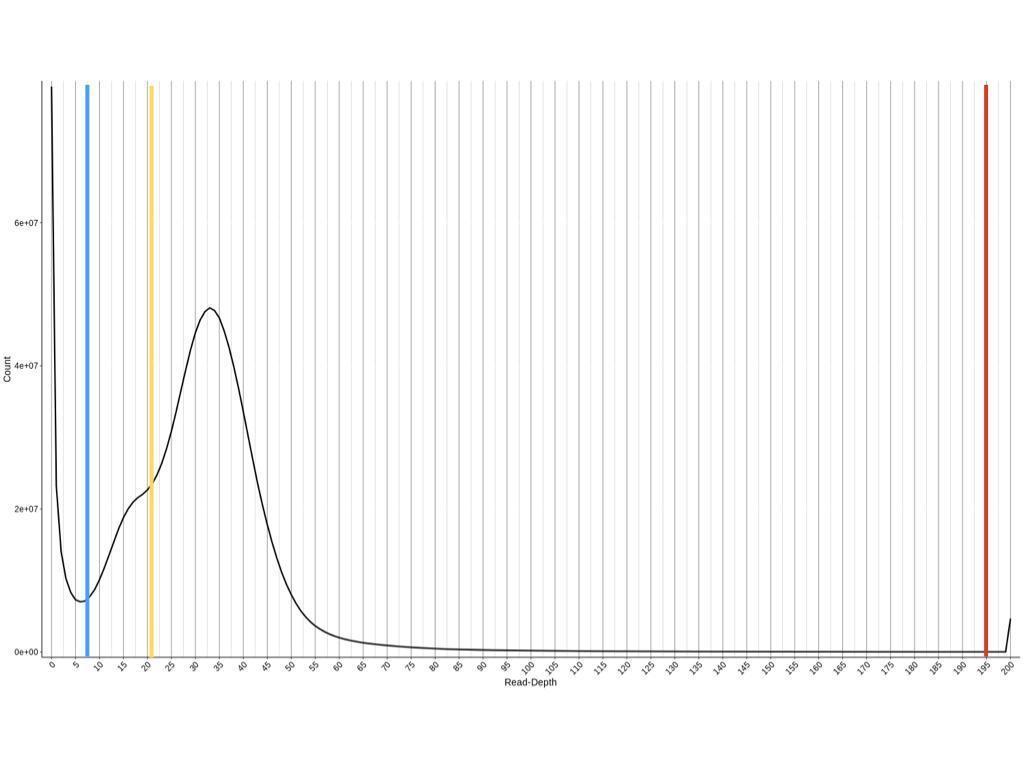


Canu


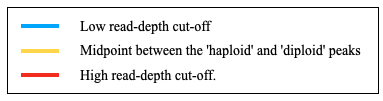


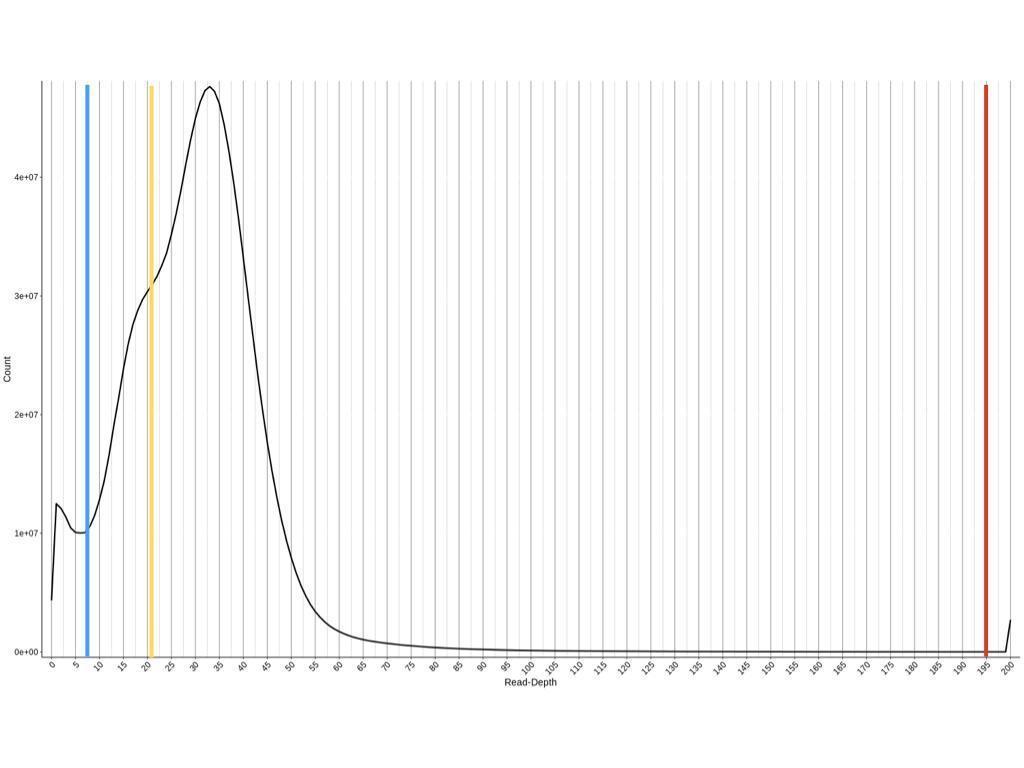


MaSuRCA-F


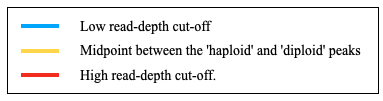


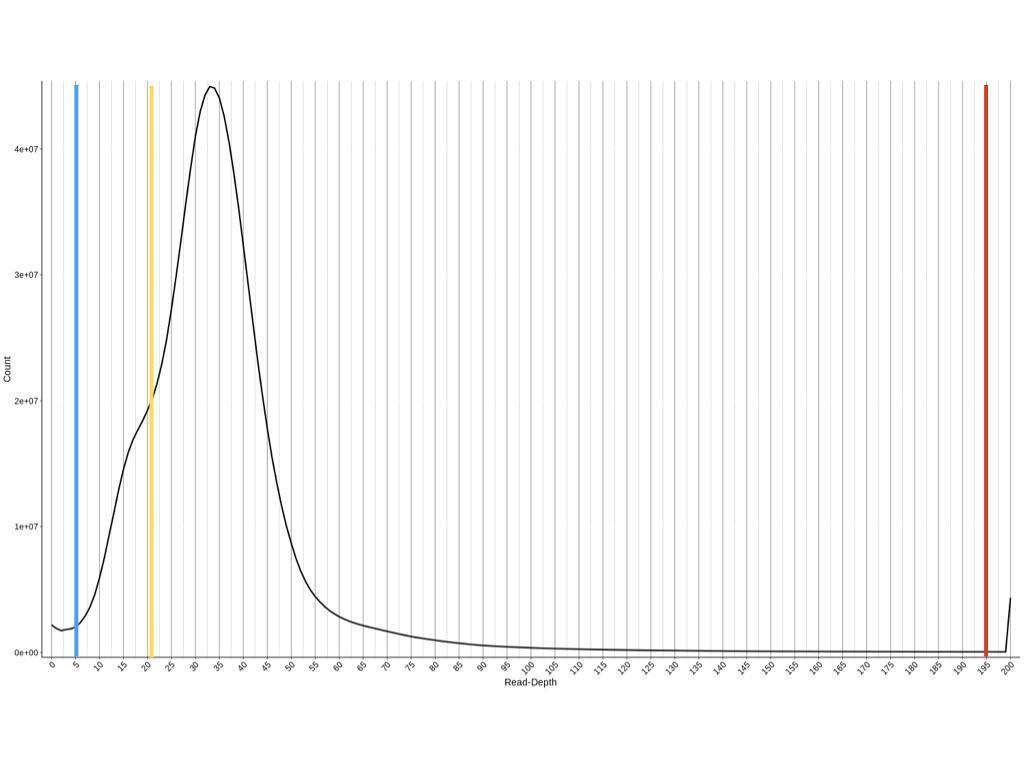


MaSuRCA-C


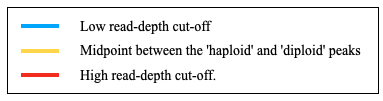


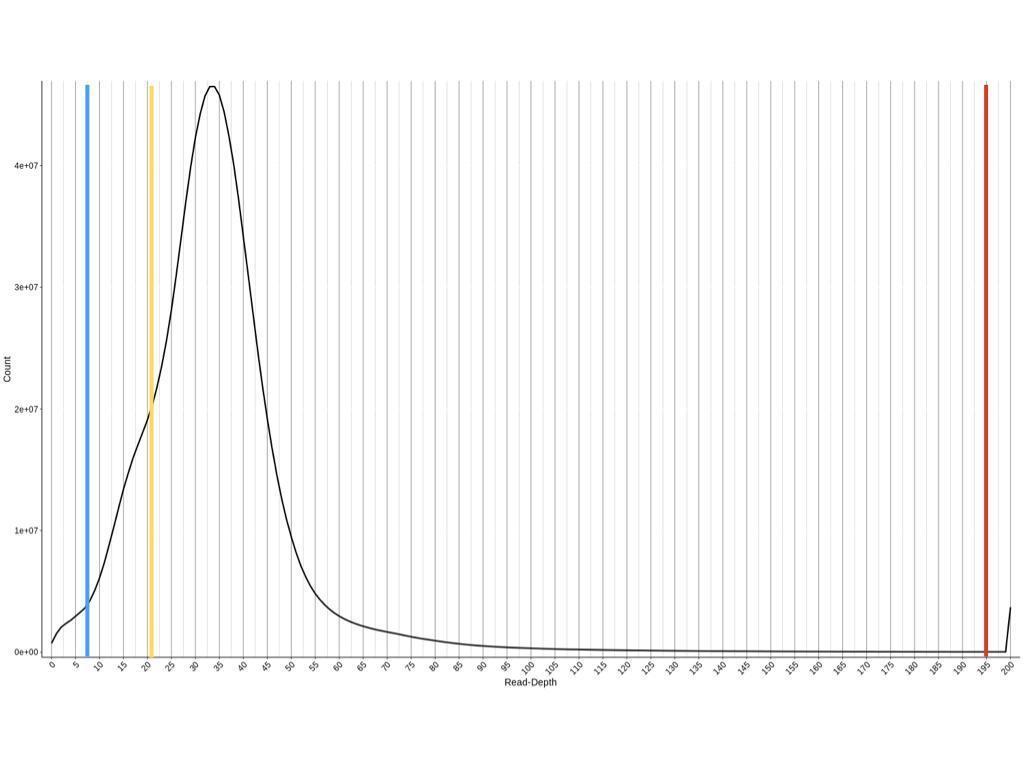


1. *A. thaliana* F1

HASLR


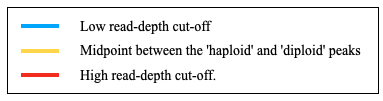


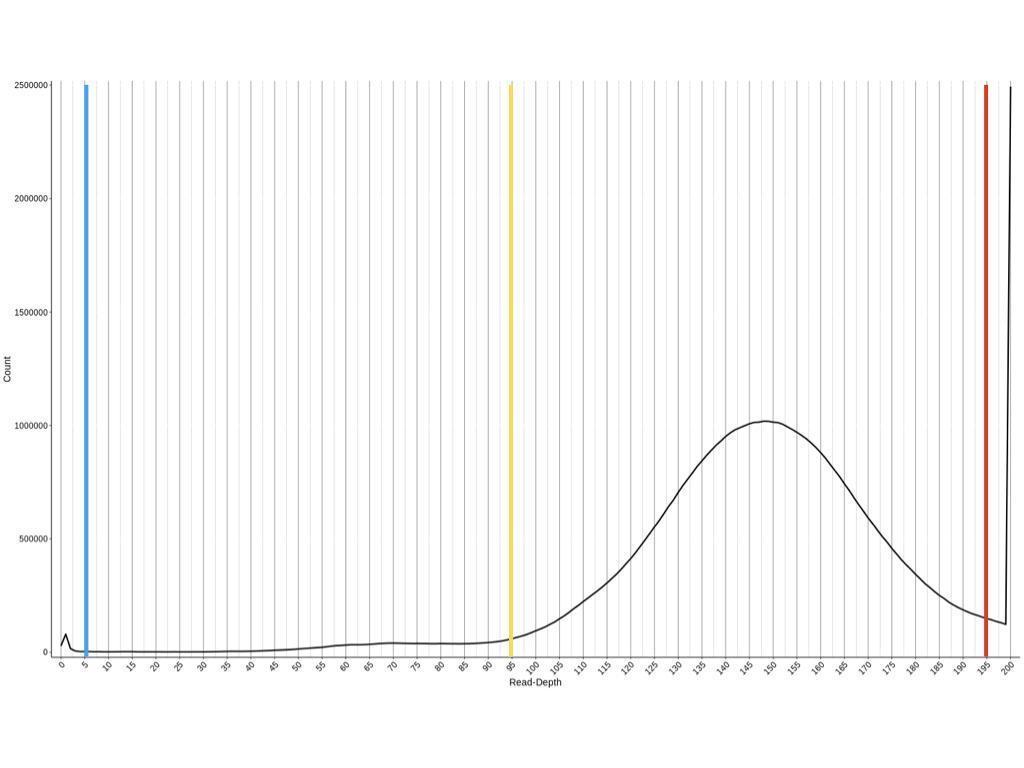


WENGAN-M


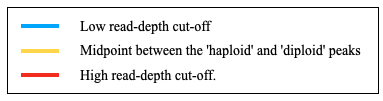


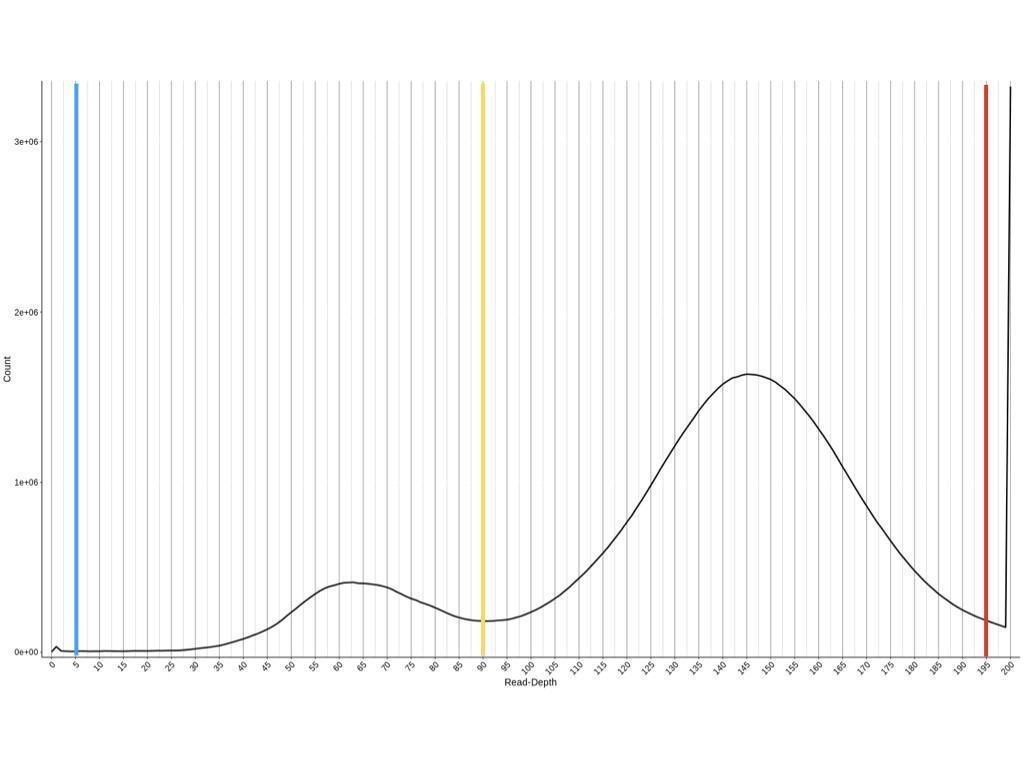


Redbean


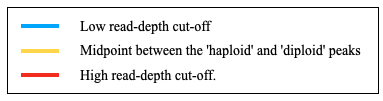


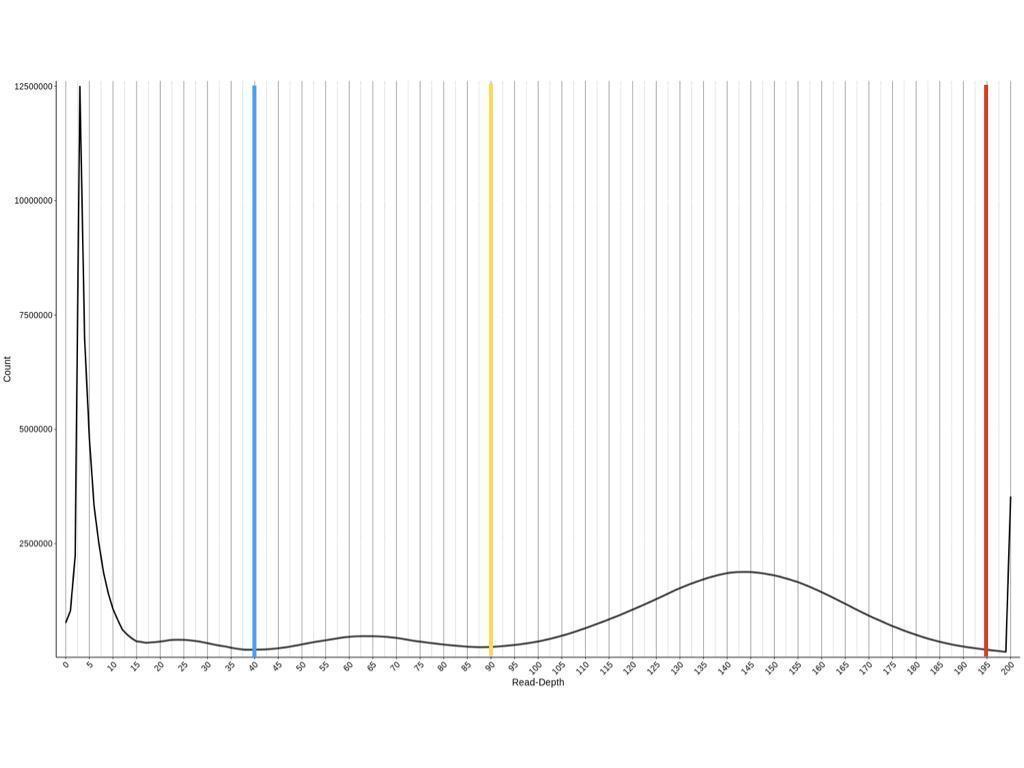


miniasm


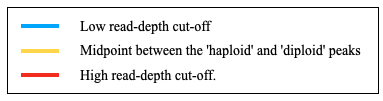


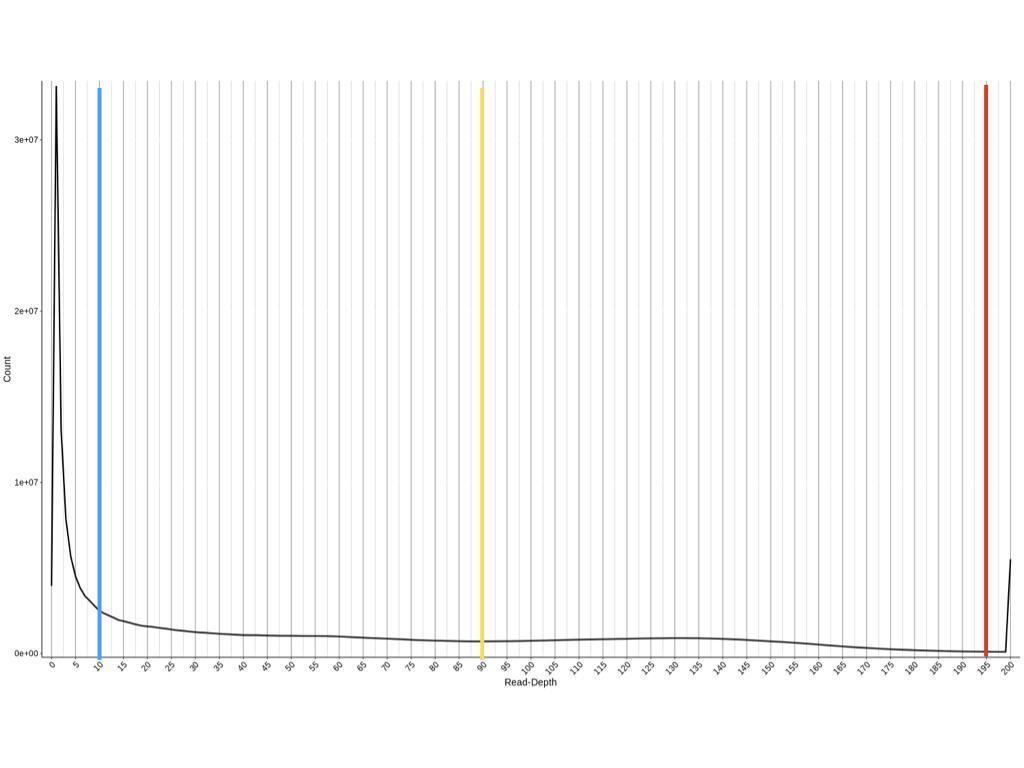


Flye


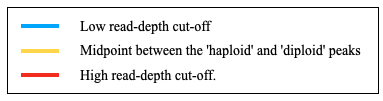


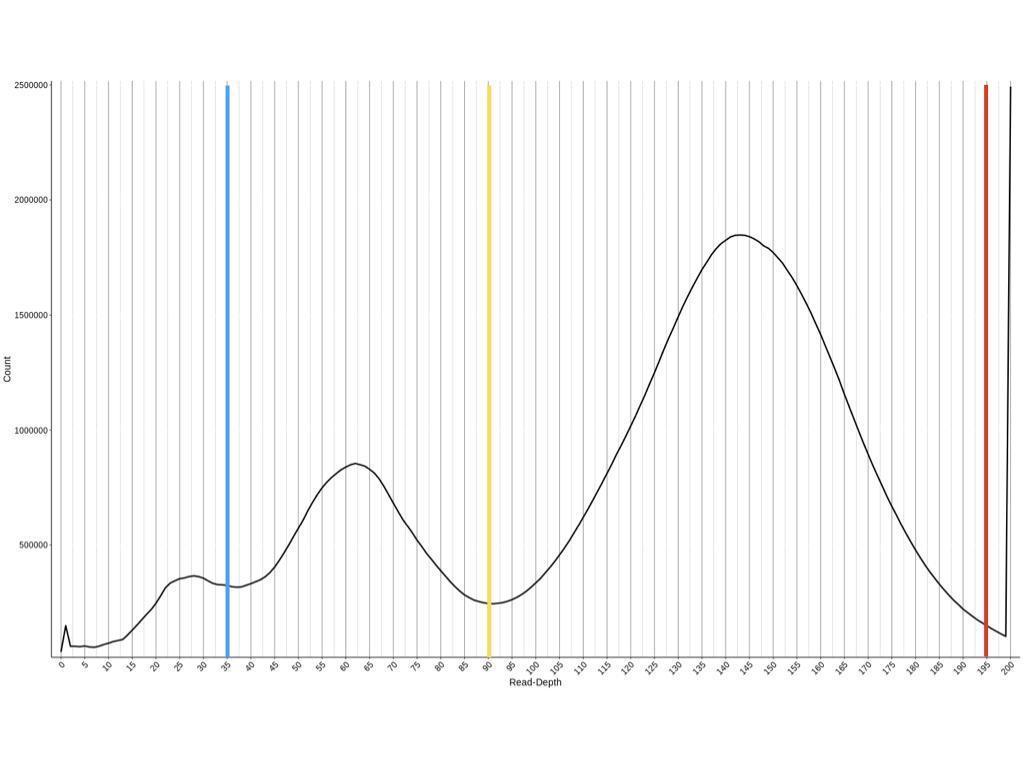


NextDenovo


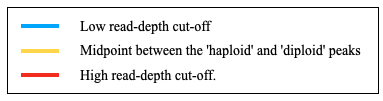


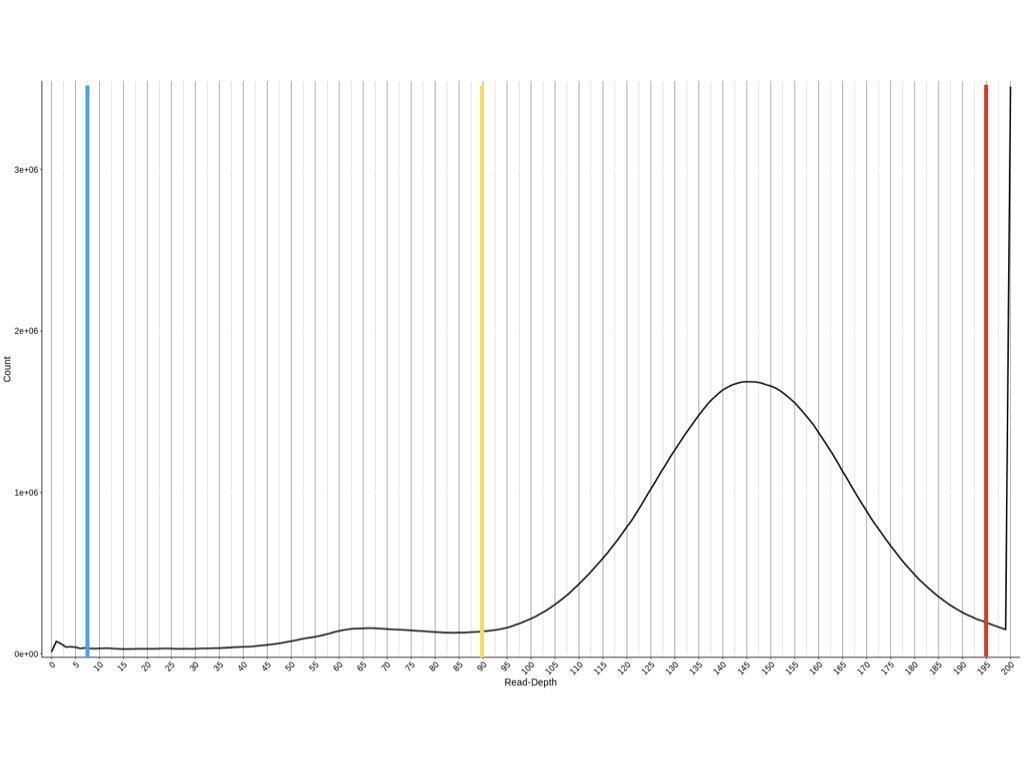


SPAdes


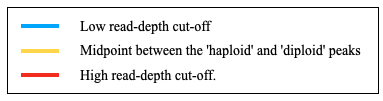


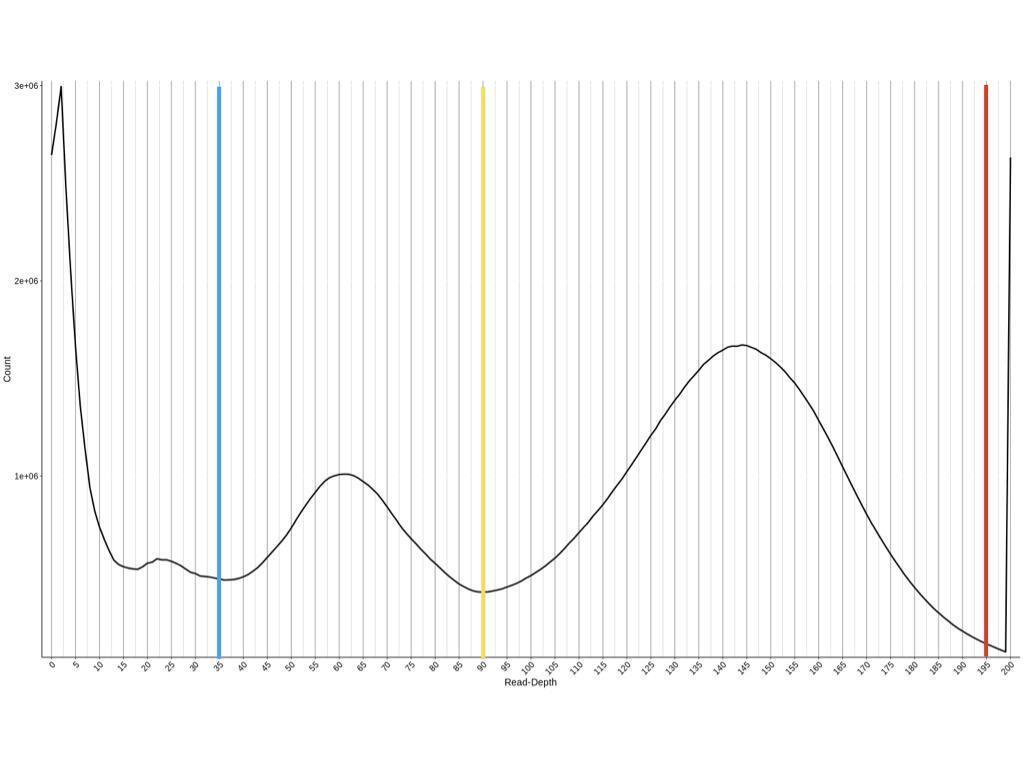


Platanus-allee

Canu

MaSuRCA-F

MaSuRCA-C

1. *C. gigas*

HASLR

WENGAN-M

Redbean

miniasm

Flye

NextDenovo

SPAdes

Platanus-allee

Canu

MaSuRCA-F

MaSuRCA-C

Figure S1. Histogram of read-depth to contigs by Purge Haplotigs.

(A) *A. thaliana* C24; (B) *N. putrida* NIES-4239; (C) *L. calcarifer*; (D) *S. sitiens*; (E) *A. thaliana* F1; (F) *C. gigas.* Vertical lines represent the read-depth cut-offs for the parameters of Purge Haplotigs. Blue line indicates low read-depth cut-off, yellow line indicates the midpoint between the 'haploid' and 'diploid' peaks, and red line indicates a high read-depth cut-off.

*A. thaliana* C24

Polished contigs

Primary contigs

*N. putrida* NIES-4239

Polished contigs

Primary contigs

*L. calcarifer*

Polished contigs

Primary contigs

*S. sitiens*

Polished contigs

Primary contigs

*A. thaliana* F1

Polished contigs

Primary contigs

*C. gigas*

Polished contigs

Primary contigs

Figure S2. Comparison of the cumulative length between polished contigs and primary contigs by Purge Haplotigs.

(A) *A. thaliana* C24 and (B) *N. putrida* NIES-4239; x-axis was cut at 1000. (C) *L. calcarifer*; x-axis was cut at 10000. Canu was timeout. (D) *S. sitiens*; x-axis was cut at 90000. (E) *A. thaliana* F1; x-axis was cut at 14000. (F) *C. gigas*; x-axis was cut at 90000. Upper graphs show the cumulative length of polished contigs and lower graphs show primary contigs by Purge Haplotigs. Estimated genome sizes are indicated by red dash lines, which are 1 of the assembly ploidy^*^. Half of the estimated genome sizes are indicated by black dash lines, which represent 0.5 of the assembly ploidy. Twice the estimated genome size is indicated by blue dash lines, which represent 1.5 of the assembly ploidy.

^*^  Assembly ploidy is a metric that indicates the expansion degree of the haploid representation, was calculated by dividing the total length by the estimated genome size.

(A) *A. thaliana* C24

(B) *N. putrida* NIES-4239

(C) *L. calcarifer*

(D) *S. sitiens*

(E) *A. thaliana* F1

(F) *C. gigas*

Figure S3. Nx statistics for polished contigs.

(A) *A. thaliana* C24; (B) *N. putrida* NIES-4239; (C) *L. calcarifer*; (D) *S. sitiens*; (E) *A. thaliana* F1; (F) C*. gigas*. Canu was timeout in (C).

(A) *A. thaliana* C24

(B) *N. putrida* NIES-4239

(C) *L. calcarifer*

(D) *S. sitiens*

(E) *A. thaliana* F1

(F) *C. gigas*

Figure S4. BUSCO completeness for primary contigs.

(A) *A. thaliana* C24; (B) *N. putrida* NIES-4239; (C) *L. calcarifer*; (D) *S. sitiens*; (E) *A. thaliana* F1; (F) *C. gigas*. Each breakdown and colour bar represent the categories of BUSCO: The entire complete, including 'single-copy' and 'duplicated', is represented by 'C'. Blue indicates 'single-completeness' represented by 'S', orange indicates 'duplicated-completeness' represented by 'D', green indicates 'fragmented' represented by 'F', and red indicates 'missing' represented by 'M'. Canu is timeout in (C).

1. *A. thaliana* C24

1. *N. putrida* NIES-4239

1. *L. calcarifer*

1. *S. sitiens*

1. *A. thaliana* F1

(F) *C. gigas*

Figure S5. BUSCO completeness for haplotigs.

(A) *A. thaliana* C24; (B) *N. putrida* NIES-4239; (C) *L. calcarifer*; (D) *S. sitiens*; (E) *A. thaliana* F1; (F) *C. gigas*. Each breakdown and colour bar represent the categories of BUSCO: The entire complete, including 'single-copy' and 'duplicated', is represented by 'C'. Blue indicates 'single-completeness' represented by 'S', orange indicates 'duplicated-completeness' represented by 'D', green indicates 'fragmented' represented by 'F', and red indicates 'missing' represented 'M'. Canu is timeout in (C).

1. *A. thaliana* C24

1. *N. putrida* NIES-4239

1. *L. calcarifer*

1. *S. sitiens*

1. *A. thaliana* F1

(F) *C. gigas*

Figure S6. BUSCO completeness for artefacts.

(A) *A. thaliana* C24; (B) *N. putrida* NIES-4239; (C) *L.* *calcarifer*; (D) *S. sitiens*; (E) *A. thaliana F1*; (F) *C. gigas*. Each breakdown and colour bar represent the categories of BUSCO: The entire complete, including 'single-copy' and 'duplicated', is represented by 'C'. Blue indicates 'single-completeness' represented by 'S', orange indicates 'duplicated-completeness' represented by 'D', green indicates 'fragmented' represented by 'F', and red indicates 'missing' represented by 'M'. Canu is timeout in (C).
