# Supplementary material for: A practical assembly guideline for genomes with various levels of heterozygosity
Source: Brief Bioinform. 2023 Oct 5;24(6):bbad337. doi: 10.1093/bib/bbad337 (PMC10555665; doi:10.1093/bib/bbad337)
Supplement: Supplementary_Methods_bbad337 [file supplementary_methods_bbad337.docx]

**Supplementary Methods**

Details of the execution commands and configuration options of each tool are described below.

**Notations in the commands**

pac_subreads.fastq.gz: PacBio subreads

illumina_1.fastq.gz: Illumina read1

illumina_2.fastq.gz: Illumina read2

illumina_1.trimmed.fastq.gz: Illumina trimmed read1 by fastp

illumina_2.trimmed.fastq.gz: Illumina trimmed read2 by fastp

assembly_prefix: assembly prefix

assembly_directory: assembly directory

polish_dir: polishing directory

contigs.fa: contigs by assembler

contigs_polished.fasta: the polished contigs

genome_size: Estimated genome size by GenomeScope

*Arabidopsis thaliana* C24: 120M

*Nitzschia putrida* NIES-4239: 30M

*Lates calcarifer*: 550M

*Solanum sitiens*: 900M

*Arabidopsis thaliana* F1: 120M

*Crassostrea gigas*: 490M

***De novo* assemblers with PacBio long reads**

**Canu v2.1.1**

canu -p assembly_prefix -d assembly_directory genomeSize=genome_size \

useGrid=false maxThreads=10 -pacbio pac_subreads.fastq.gz

**Flye v2.8.3**

flye --pacbio-raw pac_subreads.fastq.gz \

--out-dir assembly_directory --threads 10

**Redbean v2.5**

wtdbg2 -t 10 -i pac_subreads.fastq.gz -f -o assembly_prefix

wtpoa-cns -t 10 -i assembly_prefix.ctg.lay.gz -f -o assembly_prefix.ctg.lay.fa

**Miniasm v0.3-r179 (minimap2 v2.17-r941)**

minimap2 -x ava-pb -t 10 \

pac_subreads.fastq.gz pac_subreads.fastq.gz | gzip -1 > reads.paf.gz

miniasm -f pac_subreads.fastq.gz reads.paf.gz > assembly.gfa

awk '/^S/{print ">"$2"\n"$3}' assembly.gfa | fold > assembly.fa

**NextDenovo (v2.4.0)**

nextDenovo run.cfg

*Contents of run.cfg*

[General]

job_type = local

job_prefix = nextDenovo

task = all # 'all', 'correct', 'assemble'

rewrite = yes # yes/no

deltmp = yes

input_type = raw

read_type = clr

input_fofn = ./input.fofn

workdir = ./01_rundir

[correct_option]

genome_size = genome_size

sort_options = -m 40g -t 10

minimap2_options_raw = -t 10

[assemble_option]

minimap2_options_cns = -t 10

minimap2_options_map = -t 10

**Hybrid *de novo* assemblers**

Regarding the Illumina reads used for assemblies, trimmed reads were used for HASLR, Platauns-allee, SPAdes, and WENGAN, while untrimmed reads were used for MaSuRCA because it required non-trimmed raw data. The Illumina data of *A. thaliana* F1 downloaded from ENA were composed of non-trimmed (x26 genome coverage) and trimmed data, of which the former were extracted for MaSuRCA .

**HASLR (v0.8a1)**

haslr.py -t 10 -o assembly_directory -g genome_size -l $PACBIO -x pacbio \

-s illumina_1.trimmed.fastq.gz illumina_2.trimmed.fastq.gz

**MaSuRCA (v4.0.1)**

masurca masurca.cfg -o assemble.sh

*Contents of masurca.cfg*

DATA

#Illumina paired end reads supplied as <two-character prefix> <fragment mean> <fragment stdev> <forward_reads> <reverse_reads>

PE= pe fragment_mean^*1^ fragment_stdev^*1^ illumina_1.fastq.gz illumina_2.fastq.gz

PACBIO=pac_subreads.fastq.gz

END

PARAMETERS

EXTEND_JUMP_READS=0

GRAPH_KMER_SIZE = auto

USE_LINKING_MATES = 0

USE_GRID=0

LHE_COVERAGE=25

MEGA_READS_ONE_PASS=0

LIMIT_JUMP_COVERAGE = 300

CA_PARAMETERS = cgwErrorRate=0.15

CLOSE_GAPS=1

NUM_THREADS = 10

# Mandatory jellyfish hash size -- a safe value is estimated_genome_size*20

JF_SIZE = jellyfish_hash_size^*2^

SOAP_ASSEMBLY=0

FLYE_ASSEMBLY=0 (CABOG:0 Flye: 1)

END

^*1^ Illumina paired end reads <fragment mean> <fragment stdev>

*Arabidopsis thaliana* C24: 500 75

*Nitzschia putrida* NIES-4239: 300 45

*Lates calcarifer*: 500 75

*Solanum sitiens*: 600 90

*Arabidopsis thaliana* F1: 500 50

*Crassostrea gigas*: 500 75

^*2^ Jellyfish hash size -- estimated_genome_size*20

*Arabidopsis thaliana* C24: 2400000000

*Nitzschia putrida* NIES-4239: 600000000

*Lates calcarifer*: 11000000000

*Solanum sitiens*: 18000000000

*Arabidopsis thaliana* F1: 2400000000

*Crassostrea gigas*: 9800000000

**Platauns-allee v2.2.2 (minimap2 v2.17-r941)**

platanus_allee assemble -t 10 -m memory -o assembly_prefix \

-f illumina_1.trimmed.fastq.gz illumina_2.trimmed.fastq.gz \

2>assembly_prefix.assembleLog

platanus_allee phase -t 10 -o assembly_prefix -mapper minimap2 \

-c assembly_prefix_contig.fa \

-IP1 illumina_1.trimmed.fastq.gz illumina_2.trimmed.fastq.gz -p pac_subreads.fastq.gz \

2>assembly_prefix.phaseLog

platanus_allee consensus -t 10 -o assembly_prefix -mapper minimap2 \

-c assembly_prefix_consensusInput.fa \

-IP1 illumina_1.trimmed.fastq.gz illumina_2.trimmed.fastq.gz -p pac_subreads.fastq.gz \

2>assembly_prefix.consensusLog

**SPAdes v3.15.0**

spades.py \

-1 illumina_1.trimmed.fastq.gz -2 illumina_2.trimmed.fastq.gz \

--pacbio pac_subreads.fastq.gz -o assembly_prefix --threads 10 --memory memory

**WENGAN v0.2**

WENGAN-M

perl wengan.pl \

-x pacraw \

-a M \

-s illumina_1.trimmed.fastq.gz,illumina_2.trimmed.fastq.gz \

-l pac_subreads.fastq.gz \

-p assembly_prefix -t 10 -g genome_size

**Pilon v1.24 (bwa v2.2.1, samtools v1.11)**

bwa-mem2 index contigs.fa

bwa-mem2 mem -t 10 contigs.fa illumina_1.trimmed.fastq.gz illumina_1.trimmed.fastq.gz > bwa.sam

samtools view -bS bwa.sam > bwa.bam

samtools sort -@ 10 bwa.bam -o bwa.sorted.bam

samtools index bwa.sorted.bam

pilon --genomecontigs.fa --frags bwa.sorted.bam --fix indels,snps --vcf --changes --verbose --outdir polish_dir

**Purge Haplotigs ver. 1.1.1 (minimap2 v2.17-r941)**

minimap2 -t 3 -ax map-pb contigs_polished.fasta pac_subreads.fastq.gz --secondary=no \

| samtools sort -m 15G -@ 3 -o aligned.bam -T tmp.ali

purge_haplotigs hist -b aligned.bam -g contigs_polished.fasta -t 2

purge_haplotigs cov -i aligned.bam.gencov -l XXX -m YYY -h ZZZ \

-o coverage_stats.csv -j 80 -s 80

purge_haplotigs purge -g contigs_polished.fasta -c coverage_stats.csv

※ The setting values of “purge_haplotigs cov” -l , -m, and -h options are listed in Supplementary Table S1.
