# Supplementary material for: A practical assembly guideline for genomes with various levels of heterozygosity
Source: Brief Bioinform. 2023 Oct 5;24(6):bbad337. doi: 10.1093/bib/bbad337 (PMC10555665; doi:10.1093/bib/bbad337)
Supplement: Supplementary_Notes_bbad337 [file supplementary_notes_bbad337.docx]

**Supplementary Notes**

The long-read-only and hybrid assemblers assessed in this study are briefly summarized below.

There are mainly two algorithms for de novo assembly: Overlap-Layout-Consensus (OLC) and de Bruijn graph (DBG) ([1–3]). The OLC algorithm consists of three steps: (1) building the overlap graph from all pairwise read alignments (overlap step), (2) transforming the overlap graph into the assembly graph, such as string graph, and selecting the best overlap graph by removing the redundant edges (layout step), and then (3) generating the consensus sequences by searching the most likely paths in the graphs (consensus step). In the DBG method, reads are split into shorter overlapping substrings of length k, called k-mer. The DBG is a directed graph typically constructed using each k-mer as an edge connecting the two nodes representing overlaps (k-1). Contigs are reconstructed by traversing the edges in the graph.

**Canu (v2.1.1)**

Canu is a fork of the Celera assembler and performs three steps with long reads: long-read correction, trimming of suspicious regions, such as adapters, and breaking of chimeras, as well as assembly using the modified best overlap graph.

**Flye (v2.8.3)**

Flye first generates disjointigs, which represent the concatenations of multiple discrete genomic segments, from the long reads. All error-prone disjointigs are concatenated, and the repeat graph is constructed using the repetitive sequences in the concatenates. The long reads are aligned to the repeat graph, and these alignments are used to untangle the repeat graph and resolve bridged repeats. Furthermore, the unabridged repeats are resolved using the small differences between repeat copies. Finally, the contigs are generated based on the path in the graph.

**Miniasm (v0.3-r179) with minimap2 (2.17-r941)**

Miniasm adopts the OLC algorithm. The overlap stage is performed using minimap, which is the mapper for long reads, and the layout stage is implemented in miniasm. The consensus stage and the error correction step for long noisy reads are omitted. Thus, the contigs are produced ultrafast but are not of high quality.

**NextDenovo (v2.4.0)**

NextDenovo performs the error correction for long reads in the NextCorrect module and the construction of a string graph with corrected reads in the NextGraph module.

**Redbean (v2.5)**

Redbean (wtdbg2) mainly follows the OLC algorithm, but the layout algorithm is based on the ‘fuzzy’ de Bruijn graph, which is a modified DBG algorithm that allows imperfect matches for long noisy reads.

**HASLR (v0.8a1)**

HASLR first performs de novo assembly with short reads. The contigs are mapped to long reads and unique hit contigs are extracted. Then, the backbone graph, which is a model of the connections between contigs aligned on long reads that provides a large-scale map of the whole genome, is built with only unique hit contigs because multiple-hit contigs will cause branches in the backbone graph. Next, the backbone graph is simplified to reduce the effects of incorrect contigs and long read alignments. Finally, consensus sequences are generated by extracting simple paths.

**MaSuRCA (v4.0.1)**

MaSuRCA combines the benefits of DBG and OLC assembly approaches. MaSuRCA first extends short reads into super-reads, and then combines the super-reads and long reads into mega-reads. Subsequently, these mega-reads are assembled using CABOG or Flye. We benchmarked using MaSuRCA_C (CABOG) and MaSuRCA_F (Flye). Illumina mate pairs, and legacy Sanger sequence data can be used in addition to Illumina paired-end reads and long reads.

**Platanus-allee (v2.2.2) with minimap2 (2.17-r941)**

Platanus-allee is a de novo haplotype assembler. First, short reads are assembled using DBG. Unlike many other haplotype assemblers, Platanus-allee generates each haplotype sequence in a diploid genome separately during this first step. Next, the graph structure is simplified with the sequence link information, and the homologous regions are anchored using the bubble structure in the initial graph. Platanus-allee also takes syntenic conservation between a homologous chromosome pair into account to further improve structural accuracy and extend the sequence.

**SPAdes (v3.15)**

SPAdes constructs the assembly graph, which is based on the DBG algorithm, from short reads. Long reads are mapped to the assembly graph, and gaps are closed using the consensus of long reads traversing the gaps. Repeats are resolved by incorporating the long-read path, generated from the alignment between long reads and the assembly graph, into the decision rule of ExSPAnder, which is a module of SPAdes used to resolve repeats and close gaps.

**WENGAN (v0.2)**

WENGAN assembles using DBG-based de novo assemblers MINIA3 [4], ABYSS2 [5], or DISCOVERdenovo [6] and then creates multiple synthetic paired end reads with different insert sizes from long reads. These synthetic paired-ends are mapped to the short read-based contigs. Using this alignment, the Synthetic Scaffolding Graph (SSG) is built. The final contigs are generated using the SSG by traversing the optimized path, filling the gaps, and polishing. We benchmarked using WENGAN-M (MINIA3).

**References**

1. Chen Q, Lan C, Zhao L et al. Recent advances in sequence assembly: principles and applications. Brief Funct Genomics 2017;16:361–378.

2. Li FW, Harkess A. A guide to sequence your favorite plant genomes. Appl Plant Sci 2018;6:e1030.

3. Miller JR, Koren S, Sutton G. Assembly algorithms for next-generation sequencing data. Genomics 2010;95:315–327.

4. Chikhi R, Rizk G. Space-Efficient and exact de Bruijn graph representation based on a Bloom filter. Algorithms Mol Biol 2013;8:22.

5. Jackman SD, Vandervalk BP, Mohamadi H et al. ABySS 2.0: resource-efficient assembly of large genomes using a Bloom filter. Genome Res 2017;27:768–77.

6. Weisenfeld NI, Yin S, Sharpe T et al. Comprehensive variation discovery in single human genomes. Nat Genet 2014;46:1350–55.
